# Supplementary material for: Single Nucleus RNA-sequencing Reveals Altered Intercellular Communication and Dendritic Cell Activation in Nonobstructive Hypertrophic Cardiomyopathy
Source: Cardiol Cardiovasc Med. Author manuscript; Available in PMC 2022 Oct 12. (PMC9555339; doi:10.26502/fccm.92920277)
Supplement: Supply [file NIHMS1832242-supplement-Supply.pdf]

Supplemental Table 1. Biomarker list with citations

Cell type	Biomarker	Reference	Note
Cardiomyocyte	MYBPC3	Selewa et al., 2020; Litviňuková et al., 2020	
	MYH7B	Cui et al., 2019	
	MYH7	Cui et al., 2019; Wang et al., 2020; Litviňuková et al., 2020	
	TNNT2	Selewa et al., 2020; Lother et al., 2018	
	LDB3	Jia et al., 2018	
	FHOD3	Tucker et al., 2021	
	PLN	Lother et al., 2018; Litviňuková et al., 2020	Mature
	ACTC1	Wang et al., 2020; Litviňuková et al., 2020	Mature
	TNNC	McLellan et al., 2020; Jia et al., 2018	Mature
	GATA4	Jia et al., 2018; Hu et al., 2018	
	MYOCD	Jia et al., 2018; Hu et al., 2019	Developing
	MHRT	McLellan et al., 2020; Hu et al., 2019	Developing
	NPPA	Cui et al., 2019; Wang et al., 2020; Litviňuková et al., 2020	Trabecular
	MYH6	Lother et al., 2018; Cui et al., 2019	
	ATP2A2	Lother et al., 2018; Wang et al., 2020	
	RYR2	Lother et al., 2018; Litviňuková et al., 2020; Hu et al., 2019	
	MYL2	Wang et al., 2020; Litviňuková et al., 2020	
Fibroblasts	FMO2	Tucker et al., 2021	
	POSTN	Cui et al., 2019; Selewa et al., 2020; McLellan et al., 2020; Litviňuková et al., 2020	
	COL6A3	Wang et al., 2020	
	COL3A1	McLellan et al., 2020; Cui et al., 2019	
	FBN1	McLellan et al., 2020; Hu et al., 2018	
	COL1A1	Lother et al., 2018; McLellan et al., 2020; Cui et al., 2019	
	DDR2	Wang et al., 2020	
	PLEKHH2	Tucker et al., 2021	
	IGF1	Tucker et al., 2021	
	SCN7A	Poulsen et al., 2020	Also neuronal marker
	COL1A2	Cui et al., 2019	
Myofibroblasts	DES	Wang et al., 2020	Repeated in cardiomyocytes
	COL5A2	Selewa et al., 2020	
	ACTA2	Tarbit et al., 2019	Also smooth muscle
	PALLD	Tucker et al., 2021	
Endothelial	PECAM1	Lother et al., 2018; Litviňuková et al., 2020; Cui et al., 2019	
	VWF	Lother et al., 2018; Litviňuková et al., 2020	
	AQP1	Wang et al., 2020	
	CDH5	Lother et al., 2018; Litviňuková et al., 2020	
	TEK	Lother et al., 2018; Wang et al., 2020; Jia et al., 2018	
	KDR	Lother et al., 2018; Wang et al., 2020	
	SOX7	Lother et al., 2018	Venous
	NF2F2	Lother et al., 2018	Venous
Smooth Muscle	MYH11	McLellan et al., 2020; Cui et al., 2019	Classic
	ACTA2	McLellan et al., 2020; Litviňuková et al., 2020; Wang et al., 2020	Classic, repeated in myofibroblasts
	TAGLN	McLellan et al., 2020	Classic
	CNN1	Skelton et al., 2014	
	OLFR558	McLellan et al., 2020	
	LMOD1	McLellan et al., 2020	
	NRIP2	McLellan et al., 2020	
	PI16	Cui et al., 2019	Immature
	CPZ	Cui et al., 2019	Immature
	PDGFRL	Cui et al., 2019	Immature
Pericytes	PDGFRB	Tucker et al., 2021; McLellan et al., 2020	Smooth Muscle and Pericyte marker, repeated in smooth muscle
	PDGFRA	McLellan et al., 2020	Repeated in fibroblasts
	COLEC11	McLellan et al., 2020	
	ABCC9	McLellan et al., 2020	
	KCNJ8	McLellan et al., 2020	
	VTN	McLellan et al., 2020	
	STEAP4	McLellan et al., 2020	
	NOTCH3	Wang et al., 2020; Litviňuková et al., 2020	Smooth Muscle and Pericyte marker, repeated in smooth muscle
Lymphatic	LYVE1	McLellan et al., 2020	
	MMRN1	McLellan et al., 2020	
	CCL21A	McLellan et al., 2020	
	CLDN5	McLellan et al., 2020	
	FLT1	McLellan et al., 2020	
Dendritic	CD11C	Novershtern et al., 2011; Merad et al., 2013	
	HLA-DR		
Neuronal	SCN7A	Tucker et al., 2021	
	NOVA1	Tucker et al., 2021	
	VIM	Tucker et al., 2021	Schwann, repeated in myofibroblasts, fibroblasts
	ANXA1	Tucker et al., 2021	Schwann
Leukocyte	MRC1	Tucker et al., 2021	
	FYB	Tucker et al., 2021	
	CD86	Tucker et al., 2021	
Natural Killer	CD3 (not expressed)	Montaldo et al., 2013; Chen et al., 2015; Colucci et al., 2003; Farag &Caligiuri, 2006; Humana Press, 2007	
	CD56		
	CD94		
	NKp46		
T	CD3	Humana Press, 2007; Balon et al., 2006; Finak et al., 2016	
B	CD19	Humana Press, 2007; Kaminski et al., 2012; Orlic et al., 1993; Bendall et al., 2014; Wood, 2004	
	CD79A	McLellan et al., 2020	
	LY6D	McLellan et al., 2020	
	H2-DMB2	McLellan et al., 2020	
	CD79B	McLellan et al., 2020	
	MS4A1	McLellan et al., 2020	
Monocyte	CD14	Novershtern et al., 2011; Wood, 2004; Yang et al., 2014	
	PTPRC (CD45)		
	CSFR3R		
Macrophage	CD11B	Murray et al., 2011; Pilling et al., 2009	
	CD68		
	CD163		
	CSF1R	McLellan et al., 2020	
	ADGRE1	McLellan et al., 2020	
	PLD4	McLellan et al., 2020	
	MS4A6C	McLellan et al., 2020	
	MGL2	McLellan et al., 2020	
Neutrophil	CD11B	Humana Press, 2007; Wood, 2004; Elghetany & Elghetany, 2002; Mantovani et al., 2011; Behnen et al., 2014	
	CD16		
	CD18		
	CD32		
	CD55		

Supplemental Table 2. Consensus Cell Identity Assignments for Each Cluster (blue)

	Cell Assignment Method			
**Cluster**	**Biomarkers**	**pangloadb overexpressed genes**	**Gene Ontology**	**Inginuity Pathway Analysis**
**0**	Cardiomyocyte	Uknown	Cardiomyocyte	Cardiomyocyte
**1**	Cardiomyocyte	Unknown	Cardiomyocyte	Cardiomyocyte
**2**	Endothelial	Endothelial	Endothelial	Endothelial
**3**	Cardiomyocyte	Uknown	Cardiomyocyte	Cardiomyocyte
**4**	Fibroblast	Fibroblast	Unknown	Unknown
**5**	Fibroblast	Fibroblast	Fibroblast	Fibroblast
**6**	Leukocyte	Dendritic, Unknown	Leukocyte	Unknown
**7**	Cardiomyocyte	Unknown	Cardiomyocyte	Cardiomyocyte
**8**	Cardiomyocyte	Unknown	Cardiomyocyte	Cardiomyocyte
**9**	Cardiomyocyte	Unknown	Unknown	Cardiomyocyte
**10**	Pericyte	Unknown	Pericyte	Unknown
**11**	Dendritic, Leukocyte	Dendritic	Dendritic	Unknown
**12**	Fibroblast	Fibroblast	Cardiomyocyte	Unknown
**13**	Cardiomyocyte	Unknown	Cardiomyocyte	Cardiomyocyte
**14**	Cardiomyocyte	Unknown	Unknown	Cardiomyocyte
**15**	Smooth Muscle	Smooth Muscle	Smooth Muscle	Unknown
**16**	Cardiomyocyte, Neuronal	Uknown	Neuronal	Neuronal
**17**	Fibroblast	Fibroblast	Unknown	Fibroblast
**18**	Fibroblast	Fibroblast	Unknown	Unknown
**19**	Cardiomyocyte	Uknown	Unknown	Cardiomyocyte
**20**	Fibroblast	Fibroblast	Unknown	Unknown
**21**	Cardiomyocyte	Unknown	Cardiomyocyte	Unknown

Cell Type	Condition	*Number of*		
		Nuclei	Differentially expressed genes	Gene overlap
Cardiomyocyte	Normal	16659	6695	4518
	Non-obstructive	26964	4851	
Fibroblast	Normal	9147	831	206
	Non-obstructive	9251	324	
Endothelial	Normal	2214	578	164
	Non-obstructive	5491	359	
Pericyte	Normal	1351	407	48
	Non-obstructive	2260	159	
Dendritic	Normal	1558	450	51
	Non-obstructive	1604	142	
Leukocyte	Normal	2457	226	25
	Non-obstructive	1754	136	
Smooth Muscle	Normal	563	250	33
	Non-obstructive	961	173	
Neuronal	Normal	409	295	18
	Non-obstructive	725	115	

Supplemental Table 3. Number of differentially expressed genes by cell type and condition. Differentially expressed genes were determined by positive Moran's I statistic values when respective adjusted p-values were ≤ 0.05 and when genes were expressed in ≥ 1% of all cells in a cell type. The number of cells per cell class and number of overlapping differentially expressed genes among cell classes are also listed.

Supplemental Table 4. Differentially Expressed Genes in Nonobstructive HCM

	Filtered Differentially Expressed Genes Over Space	Cell Type
1	ABCA10	Cardiomyocyte, Fibroblast
2	ABCA6	Cardiomyocyte
3	ABCA8	Cardiomyocyte
4	AC010680.1	Cardiomyocyte
5	AC010680.5	Cardiomyocyte
7	ADH1B	Cardiomyocyte
8	AGT	Endothelial, Pericyte
11	B2M	Dendritic, Neuronal
12	C1R	Fibroblast
14	CARMN	Smooth Muscle
18	CD69	Dendritic
20	CLU	Endothelial
21	COL1A2	Neuronal
23	DNM3OS	Cardiomyocyte
24	EDN1	Endothelial
25	EMC10	Cardiomyocyte
27	FBLN2	Fibroblast
28	GZMA	Dendritic
30	HES4	Smooth Muscle
31	HLA-DPA1	Dendritic
33	HLA-DRB1	Dendritic
36	IGFBP7	Endothelial, Pericyte
37	LINC00861	Dendritic
38	MEG3	Cardiomyocyte, Fibroblast
39	MMRN1	Endothelial
40	MS4A6A	Dendritic
41	MYH7	Neuronal
42	MYH7B	Cardiomyocyte
43	MYL2	Cardiomyocyte
44	NDUFA4L2	Pericyte
45	NEBL	Cardiomyocyte,Neuronal
46	NPPB	Cardiomyocyte
47	PDGFRB	Pericyte, Smooth Muscle
50	RGS1	Leukocyte
51	RGS5	Endothelial, Pericyte
52	RP11-394O4.5	Smooth Muscle
53	RP11-532N4.2	Cardiomyocyte
54	SAT1	Dendritic
55	SLC8A1	Cardiomyocyte
57	SPARC	Neuronal
58	SPARCL1	Pericyte
59	SPP1	Neuronal
60	TFPI	Endothelial
61	TMSB4X	Endothelial
63	TTN	Neuronal
64	VIM	Neuronal

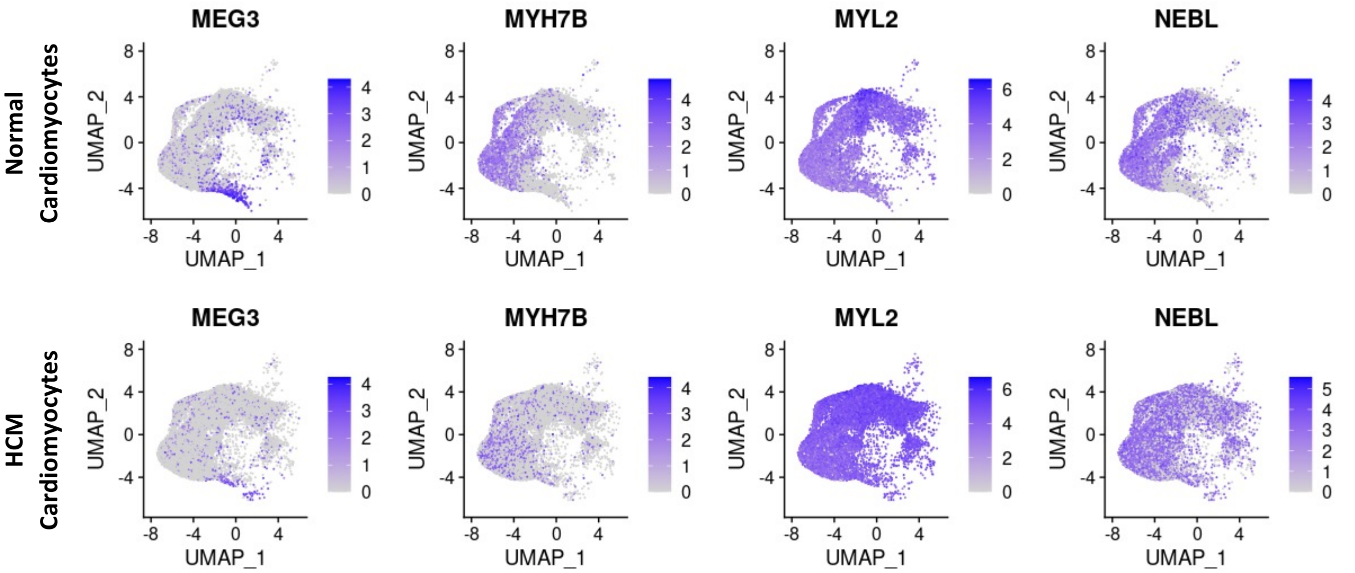

Supplemental Figure 1. Representative Differentially Expressed Genes in Cardiomyocytes, Determined by Spatial Autocorrelation, Plotted in UMAP Space

Supplemental Table 5. Increased Receptor Signaling to Dendritic Cells

Normal				
Pair_Name	Ligand	Receptor	L_cell	R_cell
COL1A2_CD36	COL1A2	CD36	CM	DC
LGALS1_PTPRC	LGALS1	PTPRC	CM	DC
TIMP1_CD63	TIMP1	CD63	CM	DC
APP_CD74	APP	CD74	Fibro	DC
COL1A1_CD36	COL1A1	CD36	Fibro	DC
COL1A2_CD36	COL1A2	CD36	Fibro	DC
LGALS1_PTPRC	LGALS1	PTPRC	Fibro	DC
TIMP1_CD63	TIMP1	CD63	Fibro	DC
APP_CD74	APP	CD74	EC	DC
COL1A1_CD36	COL1A1	CD36	EC	DC
COL1A2_CD36	COL1A2	CD36	EC	DC
LGALS1_PTPRC	LGALS1	PTPRC	EC	DC
TIMP1_CD63	TIMP1	CD63	EC	DC
COL1A2_CD36	COL1A2	CD36	PC	DC
LGALS1_PTPRC	LGALS1	PTPRC	PC	DC
TIMP1_CD63	TIMP1	CD63	PC	DC
COL1A1_CD36	COL1A1	CD36	DC	DC
COL1A2_CD36	COL1A2	CD36	DC	DC
LGALS1_PTPRC	LGALS1	PTPRC	DC	DC
TIMP1_CD63	TIMP1	CD63	DC	DC
COL1A1_CD36	COL1A1	CD36	Leuko	DC
COL1A2_CD36	COL1A2	CD36	Leuko	DC
LGALS1_PTPRC	LGALS1	PTPRC	Leuko	DC
TIMP1_CD63	TIMP1	CD63	Leuko	DC
COL1A2_CD36	COL1A2	CD36	SMC	DC
LGALS1_PTPRC	LGALS1	PTPRC	SMC	DC
TIMP1_CD63	TIMP1	CD63	SMC	DC
COL1A2_CD36	COL1A2	CD36	Neuro	DC
LGALS1_PTPRC	LGALS1	PTPRC	Neuro	DC
TIMP1_CD63	TIMP1	CD63	Neuro	DC

Non-obstructive				
Pair_Name	Ligand	Receptor	L_cell	R_cell
CALM1_RYR2	CALM1	RYR2	CM	DC
LAMA2_ITGB1	LAMA2	ITGB1	CM	DC
LGALS1_ITGB1	LGALS1	ITGB1	CM	DC
S100A1_RYR2	S100A1	RYR2	CM	DC
TGM2_ITGB1	TGM2	ITGB1	CM	DC
CALM1_RYR2	CALM1	RYR2	Fibro	DC
COL1A2_CD36	COL1A2	CD36	Fibro	DC
COL1A2_ITGB1	COL1A2	ITGB1	Fibro	DC
COL3A1_ITGB1	COL3A1	ITGB1	Fibro	DC
COL4A1_ITGB1	COL4A1	ITGB1	Fibro	DC
COL6A1_ITGB1	COL6A1	ITGB1	Fibro	DC
COL6A2_ITGB1	COL6A2	ITGB1	Fibro	DC
COL6A3_ITGB1	COL6A3	ITGB1	Fibro	DC
FN1_ITGB1	FN1	ITGB1	Fibro	DC
LAMA2_ITGB1	LAMA2	ITGB1	Fibro	DC
LGALS1_ITGB1	LGALS1	ITGB1	Fibro	DC
LUM_ITGB1	LUM	ITGB1	Fibro	DC
S100A1_RYR2	S100A1	RYR2	Fibro	DC
CALM1_RYR2	CALM1	RYR2	EC	DC
FN1_ITGB1	FN1	ITGB1	EC	DC
LGALS1_ITGB1	LGALS1	ITGB1	EC	DC
S100A1_RYR2	S100A1	RYR2	EC	DC
CALM1_RYR2	CALM1	RYR2	PC	DC
COL1A2_CD36	COL1A2	CD36	PC	DC
COL1A2_ITGB1	COL1A2	ITGB1	PC	DC
COL4A1_ITGB1	COL4A1	ITGB1	PC	DC
COL6A1_ITGB1	COL6A1	ITGB1	PC	DC
FN1_ITGB1	FN1	ITGB1	PC	DC
LGALS1_ITGB1	LGALS1	ITGB1	PC	DC
S100A1_RYR2	S100A1	RYR2	PC	DC
CALM1_RYR2	CALM1	RYR2	DC	DC
LGALS1_ITGB1	LGALS1	ITGB1	DC	DC
S100A1_RYR2	S100A1	RYR2	DC	DC
CALM1_RYR2	CALM1	RYR2	Leuko	DC
LGALS1_ITGB1	LGALS1	ITGB1	Leuko	DC
LUM_ITGB1	LUM	ITGB1	Leuko	DC
S100A1_RYR2	S100A1	RYR2	Leuko	DC
CALM1_RYR2	CALM1	RYR2	SMC	DC
COL1A2_CD36	COL1A2	CD36	SMC	DC
COL1A2_ITGB1	COL1A2	ITGB1	SMC	DC
COL4A1_ITGB1	COL4A1	ITGB1	SMC	DC
COL6A1_ITGB1	COL6A1	ITGB1	SMC	DC
COL6A2_ITGB1	COL6A2	ITGB1	SMC	DC
FN1_ITGB1	FN1	ITGB1	SMC	DC
LGALS1_ITGB1	LGALS1	ITGB1	SMC	DC
S100A1_RYR2	S100A1	RYR2	SMC	DC
CALM1_RYR2	CALM1	RYR2	Neuro	DC
LGALS1_ITGB1	LGALS1	ITGB1	Neuro	DC
S100A1_RYR2	S100A1	RYR2	Neuro	DC

Supplemental Table 6. Increased Smooth Muscle to Leukocyte Communication in Nonobstructive HCM

Normal				
**Pair_Name**	**Ligand**	**Receptor**	**L_cell**	**R_cell**
A2M_LRP1	A2M	LRP1	SMC	Leuko
COL1A2_CD36	COL1A2	CD36	SMC	Leuko
HMGB1_CD163	HMGB1	CD163	SMC	Leuko
LGALS1_PTPRC	LGALS1	PTPRC	SMC	Leuko
PSAP_LRP1	PSAP	LRP1	SMC	Leuko
TIMP1_CD63	TIMP1	CD63	SMC	Leuko

Non-obstructive				
**Pair_Name**	**Ligand**	**Receptor**	**L_cell**	**R_cell**
CALM1_RYR2	CALM1	RYR2	SMC	Leuko
COL1A2_CD36	COL1A2	CD36	SMC	Leuko
COL1A2_ITGB1	COL1A2	ITGB1	SMC	Leuko
COL4A1_ITGB1	COL4A1	ITGB1	SMC	Leuko
COL6A1_ITGB1	COL6A1	ITGB1	SMC	Leuko
COL6A2_ITGB1	COL6A2	ITGB1	SMC	Leuko
FN1_ITGB1	FN1	ITGB1	SMC	Leuko
HMGB1_CD163	HMGB1	CD163	SMC	Leuko
LGALS1_ITGB1	LGALS1	ITGB1	SMC	Leuko
S100A1_RYR2	S100A1	RYR2	SMC	Leuko

Supplemental Table 7. Fibroblast Cluster 2 Communication with Fibroblast Clusters 2-6

Normal				
Pair_Name	Ligand	Receptor	L_cell	R_cell
APP_APLP2	APP	APLP2	Fibro 2	Fibro 2
APP_LRP1	APP	LRP1	Fibro 2	Fibro 2
C3_CD81	C3	CD81	Fibro 2	Fibro 2
C3_LRP1	C3	LRP1	Fibro 2	Fibro 2
CALR_LRP1	CALR	LRP1	Fibro 2	Fibro 2
CD99_CD81	CD99	CD81	Fibro 2	Fibro 2
COL18A1_ITGB1	COL18A1	ITGB1	Fibro 2	Fibro 2
COL1A1_ITGB1	COL1A1	ITGB1	Fibro 2	Fibro 2
COL1A2_ITGB1	COL1A2	ITGB1	Fibro 2	Fibro 2
COL3A1_ITGB1	COL3A1	ITGB1	Fibro 2	Fibro 2
COL4A1_ITGB1	COL4A1	ITGB1	Fibro 2	Fibro 2
COL5A1_ITGB1	COL5A1	ITGB1	Fibro 2	Fibro 2
COL5A2_ITGB1	COL5A2	ITGB1	Fibro 2	Fibro 2
COL6A1_ITGB1	COL6A1	ITGB1	Fibro 2	Fibro 2
COL6A2_ITGB1	COL6A2	ITGB1	Fibro 2	Fibro 2
COL6A3_ITGB1	COL6A3	ITGB1	Fibro 2	Fibro 2
CTGF_LRP1	CTGF	LRP1	Fibro 2	Fibro 2
FBLN1_ITGB1	FBLN1	ITGB1	Fibro 2	Fibro 2
FBN1_ITGB1	FBN1	ITGB1	Fibro 2	Fibro 2
FN1_ITGB1	FN1	ITGB1	Fibro 2	Fibro 2
FN1_SDC2	FN1	SDC2	Fibro 2	Fibro 2
HLA-A_APLP2	HLA-A	APLP2	Fibro 2	Fibro 2
HSP90AA1_LRP1	HSP90AA1	LRP1	Fibro 2	Fibro 2
HSP90B1_LRP1	HSP90B1	LRP1	Fibro 2	Fibro 2
HSPG2_ITGB1	HSPG2	ITGB1	Fibro 2	Fibro 2
HSPG2_LRP1	HSPG2	LRP1	Fibro 2	Fibro 2
LAMA2_ITGB1	LAMA2	ITGB1	Fibro 2	Fibro 2
LAMB1_ITGB1	LAMB1	ITGB1	Fibro 2	Fibro 2
LAMC1_ITGB1	LAMC1	ITGB1	Fibro 2	Fibro 2
LGALS1_ITGB1	LGALS1	ITGB1	Fibro 2	Fibro 2
LGALS3BP_ITGB1	LGALS3BP	ITGB1	Fibro 2	Fibro 2
LRPAP1_LRP1	LRPAP1	LRP1	Fibro 2	Fibro 2
LUM_ITGB1	LUM	ITGB1	Fibro 2	Fibro 2
MFGE8_PDGFRB	MFGE8	PDGFRB	Fibro 2	Fibro 2
MMP2_SDC2	MMP2	SDC2	Fibro 2	Fibro 2
NID1_ITGB1	NID1	ITGB1	Fibro 2	Fibro 2
PSAP_LRP1	PSAP	LRP1	Fibro 2	Fibro 2
SERPINE2_LRP1	SERPINE2	LRP1	Fibro 2	Fibro 2
SERPING1_LRP1	SERPING1	LRP1	Fibro 2	Fibro 2
TFPI_LRP1	TFPI	LRP1	Fibro 2	Fibro 2
THBS2_ITGB1	THBS2	ITGB1	Fibro 2	Fibro 2
TIMP1_CD63	TIMP1	CD63	Fibro 2	Fibro 2
TIMP2_ITGB1	TIMP2	ITGB1	Fibro 2	Fibro 2
VCAN_ITGB1	VCAN	ITGB1	Fibro 2	Fibro 2
APP_LRP1	APP	LRP1	Fibro 2	Fibro 3
C3_CD81	C3	CD81	Fibro 2	Fibro 3
C3_LRP1	C3	LRP1	Fibro 2	Fibro 3
CALR_LRP1	CALR	LRP1	Fibro 2	Fibro 3
CD99_CD81	CD99	CD81	Fibro 2	Fibro 3
COL18A1_ITGB1	COL18A1	ITGB1	Fibro 2	Fibro 3
COL1A1_ITGB1	COL1A1	ITGB1	Fibro 2	Fibro 3
COL1A2_ITGB1	COL1A2	ITGB1	Fibro 2	Fibro 3
COL3A1_ITGB1	COL3A1	ITGB1	Fibro 2	Fibro 3
COL4A1_ITGB1	COL4A1	ITGB1	Fibro 2	Fibro 3
COL5A1_ITGB1	COL5A1	ITGB1	Fibro 2	Fibro 3
COL5A2_ITGB1	COL5A2	ITGB1	Fibro 2	Fibro 3
COL6A1_ITGB1	COL6A1	ITGB1	Fibro 2	Fibro 3
COL6A2_ITGB1	COL6A2	ITGB1	Fibro 2	Fibro 3
COL6A3_ITGB1	COL6A3	ITGB1	Fibro 2	Fibro 3
CTGF_LRP1	CTGF	LRP1	Fibro 2	Fibro 3
FBLN1_ITGB1	FBLN1	ITGB1	Fibro 2	Fibro 3
FBN1_ITGB1	FBN1	ITGB1	Fibro 2	Fibro 3
FN1_ITGB1	FN1	ITGB1	Fibro 2	Fibro 3
HSP90AA1_LRP1	HSP90AA1	LRP1	Fibro 2	Fibro 3
HSP90B1_LRP1	HSP90B1	LRP1	Fibro 2	Fibro 3
HSPG2_ITGB1	HSPG2	ITGB1	Fibro 2	Fibro 3
HSPG2_LRP1	HSPG2	LRP1	Fibro 2	Fibro 3
LAMA2_ITGB1	LAMA2	ITGB1	Fibro 2	Fibro 3
LAMB1_ITGB1	LAMB1	ITGB1	Fibro 2	Fibro 3
LAMC1_ITGB1	LAMC1	ITGB1	Fibro 2	Fibro 3
LGALS1_ITGB1	LGALS1	ITGB1	Fibro 2	Fibro 3
LGALS3BP_ITGB1	LGALS3BP	ITGB1	Fibro 2	Fibro 3
LRPAP1_LRP1	LRPAP1	LRP1	Fibro 2	Fibro 3
LUM_ITGB1	LUM	ITGB1	Fibro 2	Fibro 3
MFGE8_PDGFRB	MFGE8	PDGFRB	Fibro 2	Fibro 3
NID1_ITGB1	NID1	ITGB1	Fibro 2	Fibro 3
PSAP_LRP1	PSAP	LRP1	Fibro 2	Fibro 3
SERPINE2_LRP1	SERPINE2	LRP1	Fibro 2	Fibro 3
SERPING1_LRP1	SERPING1	LRP1	Fibro 2	Fibro 3
TFPI_LRP1	TFPI	LRP1	Fibro 2	Fibro 3
THBS2_ITGB1	THBS2	ITGB1	Fibro 2	Fibro 3
TIMP1_CD63	TIMP1	CD63	Fibro 2	Fibro 3
TIMP2_ITGB1	TIMP2	ITGB1	Fibro 2	Fibro 3
VCAN_ITGB1	VCAN	ITGB1	Fibro 2	Fibro 3
APP_LRP1	APP	LRP1	Fibro 2	Fibro 4
C3_CD81	C3	CD81	Fibro 2	Fibro 4
C3_LRP1	C3	LRP1	Fibro 2	Fibro 4
CALR_LRP1	CALR	LRP1	Fibro 2	Fibro 4
CD99_CD81	CD99	CD81	Fibro 2	Fibro 4
COL18A1_ITGB1	COL18A1	ITGB1	Fibro 2	Fibro 4
COL1A1_ITGB1	COL1A1	ITGB1	Fibro 2	Fibro 4
COL1A2_ITGB1	COL1A2	ITGB1	Fibro 2	Fibro 4
COL3A1_ITGB1	COL3A1	ITGB1	Fibro 2	Fibro 4
COL4A1_ITGB1	COL4A1	ITGB1	Fibro 2	Fibro 4
COL5A1_ITGB1	COL5A1	ITGB1	Fibro 2	Fibro 4
COL5A2_ITGB1	COL5A2	ITGB1	Fibro 2	Fibro 4
COL6A1_ITGB1	COL6A1	ITGB1	Fibro 2	Fibro 4
COL6A2_ITGB1	COL6A2	ITGB1	Fibro 2	Fibro 4
COL6A3_ITGB1	COL6A3	ITGB1	Fibro 2	Fibro 4
CTGF_LRP1	CTGF	LRP1	Fibro 2	Fibro 4
FBLN1_ITGB1	FBLN1	ITGB1	Fibro 2	Fibro 4
FBN1_ITGB1	FBN1	ITGB1	Fibro 2	Fibro 4
FN1_ITGB1	FN1	ITGB1	Fibro 2	Fibro 4
HSP90AA1_LRP1	HSP90AA1	LRP1	Fibro 2	Fibro 4
HSP90B1_LRP1	HSP90B1	LRP1	Fibro 2	Fibro 4
HSPG2_ITGB1	HSPG2	ITGB1	Fibro 2	Fibro 4
HSPG2_LRP1	HSPG2	LRP1	Fibro 2	Fibro 4
LAMA2_ITGB1	LAMA2	ITGB1	Fibro 2	Fibro 4
LAMB1_ITGB1	LAMB1	ITGB1	Fibro 2	Fibro 4
LAMC1_ITGB1	LAMC1	ITGB1	Fibro 2	Fibro 4
LGALS1_ITGB1	LGALS1	ITGB1	Fibro 2	Fibro 4
LGALS3BP_ITGB1	LGALS3BP	ITGB1	Fibro 2	Fibro 4
LRPAP1_LRP1	LRPAP1	LRP1	Fibro 2	Fibro 4
LUM_ITGB1	LUM	ITGB1	Fibro 2	Fibro 4
MFGE8_PDGFRB	MFGE8	PDGFRB	Fibro 2	Fibro 4
NID1_ITGB1	NID1	ITGB1	Fibro 2	Fibro 4
PSAP_LRP1	PSAP	LRP1	Fibro 2	Fibro 4
SERPINE2_LRP1	SERPINE2	LRP1	Fibro 2	Fibro 4
SERPING1_LRP1	SERPING1	LRP1	Fibro 2	Fibro 4
TFPI_LRP1	TFPI	LRP1	Fibro 2	Fibro 4
THBS2_ITGB1	THBS2	ITGB1	Fibro 2	Fibro 4
TIMP1_CD63	TIMP1	CD63	Fibro 2	Fibro 4
TIMP2_ITGB1	TIMP2	ITGB1	Fibro 2	Fibro 4
VCAN_ITGB1	VCAN	ITGB1	Fibro 2	Fibro 4
APP_LRP1	APP	LRP1	Fibro 2	Fibro 5
C3_CD81	C3	CD81	Fibro 2	Fibro 5
C3_LRP1	C3	LRP1	Fibro 2	Fibro 5
CALR_LRP1	CALR	LRP1	Fibro 2	Fibro 5
CD99_CD81	CD99	CD81	Fibro 2	Fibro 5
COL18A1_ITGB1	COL18A1	ITGB1	Fibro 2	Fibro 5
COL1A1_CD36	COL1A1	CD36	Fibro 2	Fibro 5
COL1A1_ITGB1	COL1A1	ITGB1	Fibro 2	Fibro 5
COL1A2_CD36	COL1A2	CD36	Fibro 2	Fibro 5
COL1A2_ITGB1	COL1A2	ITGB1	Fibro 2	Fibro 5
COL3A1_ITGB1	COL3A1	ITGB1	Fibro 2	Fibro 5
COL4A1_ITGB1	COL4A1	ITGB1	Fibro 2	Fibro 5
COL5A1_ITGB1	COL5A1	ITGB1	Fibro 2	Fibro 5
COL5A2_ITGB1	COL5A2	ITGB1	Fibro 2	Fibro 5
COL6A1_ITGB1	COL6A1	ITGB1	Fibro 2	Fibro 5
COL6A2_ITGB1	COL6A2	ITGB1	Fibro 2	Fibro 5
COL6A3_ITGB1	COL6A3	ITGB1	Fibro 2	Fibro 5
CTGF_LRP1	CTGF	LRP1	Fibro 2	Fibro 5
FBLN1_ITGB1	FBLN1	ITGB1	Fibro 2	Fibro 5
FBN1_ITGB1	FBN1	ITGB1	Fibro 2	Fibro 5
FN1_ITGB1	FN1	ITGB1	Fibro 2	Fibro 5
HSP90AA1_LRP1	HSP90AA1	LRP1	Fibro 2	Fibro 5
HSP90B1_LRP1	HSP90B1	LRP1	Fibro 2	Fibro 5
HSPG2_ITGB1	HSPG2	ITGB1	Fibro 2	Fibro 5
HSPG2_LRP1	HSPG2	LRP1	Fibro 2	Fibro 5
LAMA2_ITGB1	LAMA2	ITGB1	Fibro 2	Fibro 5
LAMB1_ITGB1	LAMB1	ITGB1	Fibro 2	Fibro 5
LAMC1_ITGB1	LAMC1	ITGB1	Fibro 2	Fibro 5
LGALS1_ITGB1	LGALS1	ITGB1	Fibro 2	Fibro 5
LGALS3BP_ITGB1	LGALS3BP	ITGB1	Fibro 2	Fibro 5
LRPAP1_LRP1	LRPAP1	LRP1	Fibro 2	Fibro 5
LUM_ITGB1	LUM	ITGB1	Fibro 2	Fibro 5
MFGE8_PDGFRB	MFGE8	PDGFRB	Fibro 2	Fibro 5
NID1_ITGB1	NID1	ITGB1	Fibro 2	Fibro 5
PSAP_LRP1	PSAP	LRP1	Fibro 2	Fibro 5
SERPINE2_LRP1	SERPINE2	LRP1	Fibro 2	Fibro 5
SERPING1_LRP1	SERPING1	LRP1	Fibro 2	Fibro 5
TFPI_LRP1	TFPI	LRP1	Fibro 2	Fibro 5
THBS2_CD36	THBS2	CD36	Fibro 2	Fibro 5
THBS2_ITGB1	THBS2	ITGB1	Fibro 2	Fibro 5
TIMP1_CD63	TIMP1	CD63	Fibro 2	Fibro 5
TIMP2_ITGB1	TIMP2	ITGB1	Fibro 2	Fibro 5
VCAN_ITGB1	VCAN	ITGB1	Fibro 2	Fibro 5
APP_LRP1	APP	LRP1	Fibro 2	Fibro 6
C3_CD81	C3	CD81	Fibro 2	Fibro 6
C3_LRP1	C3	LRP1	Fibro 2	Fibro 6
CALR_LRP1	CALR	LRP1	Fibro 2	Fibro 6
CD99_CD81	CD99	CD81	Fibro 2	Fibro 6
COL18A1_ITGB1	COL18A1	ITGB1	Fibro 2	Fibro 6
COL1A1_ITGB1	COL1A1	ITGB1	Fibro 2	Fibro 6
COL1A2_ITGB1	COL1A2	ITGB1	Fibro 2	Fibro 6
COL3A1_ITGB1	COL3A1	ITGB1	Fibro 2	Fibro 6
COL4A1_ITGB1	COL4A1	ITGB1	Fibro 2	Fibro 6
COL5A1_ITGB1	COL5A1	ITGB1	Fibro 2	Fibro 6
COL5A2_ITGB1	COL5A2	ITGB1	Fibro 2	Fibro 6
COL6A1_ITGB1	COL6A1	ITGB1	Fibro 2	Fibro 6
COL6A2_ITGB1	COL6A2	ITGB1	Fibro 2	Fibro 6
COL6A3_ITGB1	COL6A3	ITGB1	Fibro 2	Fibro 6
CTGF_LRP1	CTGF	LRP1	Fibro 2	Fibro 6
FBLN1_ITGB1	FBLN1	ITGB1	Fibro 2	Fibro 6
FBN1_ITGB1	FBN1	ITGB1	Fibro 2	Fibro 6
FN1_ITGB1	FN1	ITGB1	Fibro 2	Fibro 6
HSP90AA1_LRP1	HSP90AA1	LRP1	Fibro 2	Fibro 6
HSP90B1_LRP1	HSP90B1	LRP1	Fibro 2	Fibro 6
HSPG2_ITGB1	HSPG2	ITGB1	Fibro 2	Fibro 6
HSPG2_LRP1	HSPG2	LRP1	Fibro 2	Fibro 6
LAMA2_ITGB1	LAMA2	ITGB1	Fibro 2	Fibro 6
LAMB1_ITGB1	LAMB1	ITGB1	Fibro 2	Fibro 6
LAMC1_ITGB1	LAMC1	ITGB1	Fibro 2	Fibro 6
LGALS1_ITGB1	LGALS1	ITGB1	Fibro 2	Fibro 6
LGALS3BP_ITGB1	LGALS3BP	ITGB1	Fibro 2	Fibro 6
LRPAP1_LRP1	LRPAP1	LRP1	Fibro 2	Fibro 6
LUM_ITGB1	LUM	ITGB1	Fibro 2	Fibro 6
MFGE8_PDGFRB	MFGE8	PDGFRB	Fibro 2	Fibro 6
NID1_ITGB1	NID1	ITGB1	Fibro 2	Fibro 6
PSAP_LRP1	PSAP	LRP1	Fibro 2	Fibro 6
SERPINE2_LRP1	SERPINE2	LRP1	Fibro 2	Fibro 6
SERPING1_LRP1	SERPING1	LRP1	Fibro 2	Fibro 6
TFPI_LRP1	TFPI	LRP1	Fibro 2	Fibro 6
THBS2_ITGB1	THBS2	ITGB1	Fibro 2	Fibro 6
TIMP1_CD63	TIMP1	CD63	Fibro 2	Fibro 6
TIMP2_ITGB1	TIMP2	ITGB1	Fibro 2	Fibro 6
VCAN_ITGB1	VCAN	ITGB1	Fibro 2	Fibro 6

Non-obstructive				
Pair_Name	Ligand	Receptor	L_cell	R_cell
A2M_LRP1	A2M	LRP1	Fibro 2	Fibro 2
CALM1_RYR2	CALM1	RYR2	Fibro 2	Fibro 2
CCN2_LRP1	CCN2	LRP1	Fibro 2	Fibro 2
COL1A1_CD36	COL1A1	CD36	Fibro 2	Fibro 2
COL1A1_ITGB1	COL1A1	ITGB1	Fibro 2	Fibro 2
COL1A2_CD36	COL1A2	CD36	Fibro 2	Fibro 2
COL1A2_ITGB1	COL1A2	ITGB1	Fibro 2	Fibro 2
COL3A1_ITGB1	COL3A1	ITGB1	Fibro 2	Fibro 2
COL4A1_ITGB1	COL4A1	ITGB1	Fibro 2	Fibro 2
COL6A1_ITGB1	COL6A1	ITGB1	Fibro 2	Fibro 2
COL6A2_ITGB1	COL6A2	ITGB1	Fibro 2	Fibro 2
COL6A3_ITGB1	COL6A3	ITGB1	Fibro 2	Fibro 2
FN1_ITGB1	FN1	ITGB1	Fibro 2	Fibro 2
HSP90AA1_LRP1	HSP90AA1	LRP1	Fibro 2	Fibro 2
HSP90B1_LRP1	HSP90B1	LRP1	Fibro 2	Fibro 2
LAMA2_ITGB1	LAMA2	ITGB1	Fibro 2	Fibro 2
LAMB1_ITGB1	LAMB1	ITGB1	Fibro 2	Fibro 2
LGALS1_ITGB1	LGALS1	ITGB1	Fibro 2	Fibro 2
LPL_LRP1	LPL	LRP1	Fibro 2	Fibro 2
LUM_ITGB1	LUM	ITGB1	Fibro 2	Fibro 2
PSAP_LRP1	PSAP	LRP1	Fibro 2	Fibro 2
S100A1_RYR2	S100A1	RYR2	Fibro 2	Fibro 2
TIMP1_CD63	TIMP1	CD63	Fibro 2	Fibro 2
TIMP2_ITGB1	TIMP2	ITGB1	Fibro 2	Fibro 2
VCAN_ITGB1	VCAN	ITGB1	Fibro 2	Fibro 2
CALM1_RYR2	CALM1	RYR2	Fibro 2	Fibro 3
COL1A1_CD36	COL1A1	CD36	Fibro 2	Fibro 3
COL1A1_ITGB1	COL1A1	ITGB1	Fibro 2	Fibro 3
COL1A2_CD36	COL1A2	CD36	Fibro 2	Fibro 3
COL1A2_ITGB1	COL1A2	ITGB1	Fibro 2	Fibro 3
COL3A1_ITGB1	COL3A1	ITGB1	Fibro 2	Fibro 3
COL4A1_ITGB1	COL4A1	ITGB1	Fibro 2	Fibro 3
COL6A1_ITGB1	COL6A1	ITGB1	Fibro 2	Fibro 3
COL6A2_ITGB1	COL6A2	ITGB1	Fibro 2	Fibro 3
COL6A3_ITGB1	COL6A3	ITGB1	Fibro 2	Fibro 3
FN1_ITGB1	FN1	ITGB1	Fibro 2	Fibro 3
LAMA2_ITGB1	LAMA2	ITGB1	Fibro 2	Fibro 3
LAMB1_ITGB1	LAMB1	ITGB1	Fibro 2	Fibro 3
LGALS1_ITGB1	LGALS1	ITGB1	Fibro 2	Fibro 3
LUM_ITGB1	LUM	ITGB1	Fibro 2	Fibro 3
S100A1_RYR2	S100A1	RYR2	Fibro 2	Fibro 3
TIMP1_CD63	TIMP1	CD63	Fibro 2	Fibro 3
TIMP2_ITGB1	TIMP2	ITGB1	Fibro 2	Fibro 3
VCAN_ITGB1	VCAN	ITGB1	Fibro 2	Fibro 3
CALM1_RYR2	CALM1	RYR2	Fibro 2	Fibro 4
COL1A1_CD36	COL1A1	CD36	Fibro 2	Fibro 4
COL1A1_ITGB1	COL1A1	ITGB1	Fibro 2	Fibro 4
COL1A2_CD36	COL1A2	CD36	Fibro 2	Fibro 4
COL1A2_ITGB1	COL1A2	ITGB1	Fibro 2	Fibro 4
COL3A1_ITGB1	COL3A1	ITGB1	Fibro 2	Fibro 4
COL4A1_ITGB1	COL4A1	ITGB1	Fibro 2	Fibro 4
COL6A1_ITGB1	COL6A1	ITGB1	Fibro 2	Fibro 4
COL6A2_ITGB1	COL6A2	ITGB1	Fibro 2	Fibro 4
COL6A3_ITGB1	COL6A3	ITGB1	Fibro 2	Fibro 4
FN1_ITGB1	FN1	ITGB1	Fibro 2	Fibro 4
LAMA2_ITGB1	LAMA2	ITGB1	Fibro 2	Fibro 4
LAMB1_ITGB1	LAMB1	ITGB1	Fibro 2	Fibro 4
LGALS1_ITGB1	LGALS1	ITGB1	Fibro 2	Fibro 4
LUM_ITGB1	LUM	ITGB1	Fibro 2	Fibro 4
S100A1_RYR2	S100A1	RYR2	Fibro 2	Fibro 4
TIMP1_CD63	TIMP1	CD63	Fibro 2	Fibro 4
TIMP2_ITGB1	TIMP2	ITGB1	Fibro 2	Fibro 4
VCAN_ITGB1	VCAN	ITGB1	Fibro 2	Fibro 4
CALM1_RYR2	CALM1	RYR2	Fibro 2	Fibro 5
COL1A1_CD36	COL1A1	CD36	Fibro 2	Fibro 5
COL1A1_ITGB1	COL1A1	ITGB1	Fibro 2	Fibro 5
COL1A2_CD36	COL1A2	CD36	Fibro 2	Fibro 5
COL1A2_ITGB1	COL1A2	ITGB1	Fibro 2	Fibro 5
COL3A1_ITGB1	COL3A1	ITGB1	Fibro 2	Fibro 5
COL4A1_ITGB1	COL4A1	ITGB1	Fibro 2	Fibro 5
COL6A1_ITGB1	COL6A1	ITGB1	Fibro 2	Fibro 5
COL6A2_ITGB1	COL6A2	ITGB1	Fibro 2	Fibro 5
COL6A3_ITGB1	COL6A3	ITGB1	Fibro 2	Fibro 5
FN1_ITGB1	FN1	ITGB1	Fibro 2	Fibro 5
LAMA2_ITGB1	LAMA2	ITGB1	Fibro 2	Fibro 5
LAMB1_ITGB1	LAMB1	ITGB1	Fibro 2	Fibro 5
LGALS1_ITGB1	LGALS1	ITGB1	Fibro 2	Fibro 5
LUM_ITGB1	LUM	ITGB1	Fibro 2	Fibro 5
S100A1_RYR2	S100A1	RYR2	Fibro 2	Fibro 5
TIMP1_CD63	TIMP1	CD63	Fibro 2	Fibro 5
TIMP2_ITGB1	TIMP2	ITGB1	Fibro 2	Fibro 5
VCAN_ITGB1	VCAN	ITGB1	Fibro 2	Fibro 5
CALM1_RYR2	CALM1	RYR2	Fibro 2	Fibro 6
COL1A1_CD36	COL1A1	CD36	Fibro 2	Fibro 6
COL1A1_ITGB1	COL1A1	ITGB1	Fibro 2	Fibro 6
COL1A2_CD36	COL1A2	CD36	Fibro 2	Fibro 6
COL1A2_ITGB1	COL1A2	ITGB1	Fibro 2	Fibro 6
COL3A1_ITGB1	COL3A1	ITGB1	Fibro 2	Fibro 6
COL4A1_ITGB1	COL4A1	ITGB1	Fibro 2	Fibro 6
COL6A1_ITGB1	COL6A1	ITGB1	Fibro 2	Fibro 6
COL6A2_ITGB1	COL6A2	ITGB1	Fibro 2	Fibro 6
COL6A3_ITGB1	COL6A3	ITGB1	Fibro 2	Fibro 6
FN1_ITGB1	FN1	ITGB1	Fibro 2	Fibro 6
LAMA2_ITGB1	LAMA2	ITGB1	Fibro 2	Fibro 6
LAMB1_ITGB1	LAMB1	ITGB1	Fibro 2	Fibro 6
LGALS1_ITGB1	LGALS1	ITGB1	Fibro 2	Fibro 6
LUM_ITGB1	LUM	ITGB1	Fibro 2	Fibro 6
S100A1_RYR2	S100A1	RYR2	Fibro 2	Fibro 6
TIMP1_CD63	TIMP1	CD63	Fibro 2	Fibro 6
TIMP2_ITGB1	TIMP2	ITGB1	Fibro 2	Fibro 6
VCAN_ITGB1	VCAN	ITGB1	Fibro 2	Fibro 6

Supplemental Table 8. Fibroblast Cluster 5 Communication with Fibroblast Clusters 2-6

Normal				
Pair_Name	Ligand	Receptor	L_cell	R_cell
APP_LRP1	APP	LRP1	Fibro 5	Fibro 2
COL1A1_ITGB1	COL1A1	ITGB1	Fibro 5	Fibro 2
COL1A2_ITGB1	COL1A2	ITGB1	Fibro 5	Fibro 2
COL3A1_ITGB1	COL3A1	ITGB1	Fibro 5	Fibro 2
COL6A1_ITGB1	COL6A1	ITGB1	Fibro 5	Fibro 2
COL6A2_ITGB1	COL6A2	ITGB1	Fibro 5	Fibro 2
COL6A3_ITGB1	COL6A3	ITGB1	Fibro 5	Fibro 2
FBLN1_ITGB1	FBLN1	ITGB1	Fibro 5	Fibro 2
FBN1_ITGB1	FBN1	ITGB1	Fibro 5	Fibro 2
FN1_ITGB1	FN1	ITGB1	Fibro 5	Fibro 2
FN1_SDC2	FN1	SDC2	Fibro 5	Fibro 2
HLA-A_APLP2	HLA-A	APLP2	Fibro 5	Fibro 2
HSPG2_ITGB1	HSPG2	ITGB1	Fibro 5	Fibro 2
HSPG2_LRP1	HSPG2	LRP1	Fibro 5	Fibro 2
LAMA2_ITGB1	LAMA2	ITGB1	Fibro 5	Fibro 2
LAMC1_ITGB1	LAMC1	ITGB1	Fibro 5	Fibro 2
LGALS1_ITGB1	LGALS1	ITGB1	Fibro 5	Fibro 2
LUM_ITGB1	LUM	ITGB1	Fibro 5	Fibro 2
MFGE8_PDGFRB	MFGE8	PDGFRB	Fibro 5	Fibro 2
MMP2_SDC2	MMP2	SDC2	Fibro 5	Fibro 2
PSAP_LRP1	PSAP	LRP1	Fibro 5	Fibro 2
SERPINE2_LRP1	SERPINE2	LRP1	Fibro 5	Fibro 2
SERPING1_LRP1	SERPING1	LRP1	Fibro 5	Fibro 2
TFPI_LRP1	TFPI	LRP1	Fibro 5	Fibro 2
TIMP1_CD63	TIMP1	CD63	Fibro 5	Fibro 2
VCAN_ITGB1	VCAN	ITGB1	Fibro 5	Fibro 2
APP_LRP1	APP	LRP1	Fibro 5	Fibro 3
COL1A1_ITGB1	COL1A1	ITGB1	Fibro 5	Fibro 3
COL1A2_ITGB1	COL1A2	ITGB1	Fibro 5	Fibro 3
COL3A1_ITGB1	COL3A1	ITGB1	Fibro 5	Fibro 3
COL6A1_ITGB1	COL6A1	ITGB1	Fibro 5	Fibro 3
COL6A2_ITGB1	COL6A2	ITGB1	Fibro 5	Fibro 3
COL6A3_ITGB1	COL6A3	ITGB1	Fibro 5	Fibro 3
FBLN1_ITGB1	FBLN1	ITGB1	Fibro 5	Fibro 3
FBN1_ITGB1	FBN1	ITGB1	Fibro 5	Fibro 3
FN1_ITGB1	FN1	ITGB1	Fibro 5	Fibro 3
HSPG2_ITGB1	HSPG2	ITGB1	Fibro 5	Fibro 3
HSPG2_LRP1	HSPG2	LRP1	Fibro 5	Fibro 3
LAMA2_ITGB1	LAMA2	ITGB1	Fibro 5	Fibro 3
LAMC1_ITGB1	LAMC1	ITGB1	Fibro 5	Fibro 3
LGALS1_ITGB1	LGALS1	ITGB1	Fibro 5	Fibro 3
LUM_ITGB1	LUM	ITGB1	Fibro 5	Fibro 3
MFGE8_PDGFRB	MFGE8	PDGFRB	Fibro 5	Fibro 3
PSAP_LRP1	PSAP	LRP1	Fibro 5	Fibro 3
SERPINE2_LRP1	SERPINE2	LRP1	Fibro 5	Fibro 3
SERPING1_LRP1	SERPING1	LRP1	Fibro 5	Fibro 3
TFPI_LRP1	TFPI	LRP1	Fibro 5	Fibro 3
TIMP1_CD63	TIMP1	CD63	Fibro 5	Fibro 3
VCAN_ITGB1	VCAN	ITGB1	Fibro 5	Fibro 3
APP_LRP1	APP	LRP1	Fibro 5	Fibro 4
COL1A1_ITGB1	COL1A1	ITGB1	Fibro 5	Fibro 4
COL1A2_ITGB1	COL1A2	ITGB1	Fibro 5	Fibro 4
COL3A1_ITGB1	COL3A1	ITGB1	Fibro 5	Fibro 4
COL6A1_ITGB1	COL6A1	ITGB1	Fibro 5	Fibro 4
COL6A2_ITGB1	COL6A2	ITGB1	Fibro 5	Fibro 4
COL6A3_ITGB1	COL6A3	ITGB1	Fibro 5	Fibro 4
FBLN1_ITGB1	FBLN1	ITGB1	Fibro 5	Fibro 4
FBN1_ITGB1	FBN1	ITGB1	Fibro 5	Fibro 4
FN1_ITGB1	FN1	ITGB1	Fibro 5	Fibro 4
HSPG2_ITGB1	HSPG2	ITGB1	Fibro 5	Fibro 4
HSPG2_LRP1	HSPG2	LRP1	Fibro 5	Fibro 4
LAMA2_ITGB1	LAMA2	ITGB1	Fibro 5	Fibro 4
LAMC1_ITGB1	LAMC1	ITGB1	Fibro 5	Fibro 4
LGALS1_ITGB1	LGALS1	ITGB1	Fibro 5	Fibro 4
LUM_ITGB1	LUM	ITGB1	Fibro 5	Fibro 4
MFGE8_PDGFRB	MFGE8	PDGFRB	Fibro 5	Fibro 4
PSAP_LRP1	PSAP	LRP1	Fibro 5	Fibro 4
SERPINE2_LRP1	SERPINE2	LRP1	Fibro 5	Fibro 4
SERPING1_LRP1	SERPING1	LRP1	Fibro 5	Fibro 4
TFPI_LRP1	TFPI	LRP1	Fibro 5	Fibro 4
TIMP1_CD63	TIMP1	CD63	Fibro 5	Fibro 4
VCAN_ITGB1	VCAN	ITGB1	Fibro 5	Fibro 4
APP_LRP1	APP	LRP1	Fibro 5	Fibro 5
COL1A1_CD36	COL1A1	CD36	Fibro 5	Fibro 5
COL1A1_ITGB1	COL1A1	ITGB1	Fibro 5	Fibro 5
COL1A2_CD36	COL1A2	CD36	Fibro 5	Fibro 5
COL1A2_ITGB1	COL1A2	ITGB1	Fibro 5	Fibro 5
COL3A1_ITGB1	COL3A1	ITGB1	Fibro 5	Fibro 5
COL6A1_ITGB1	COL6A1	ITGB1	Fibro 5	Fibro 5
COL6A2_ITGB1	COL6A2	ITGB1	Fibro 5	Fibro 5
COL6A3_ITGB1	COL6A3	ITGB1	Fibro 5	Fibro 5
FBLN1_ITGB1	FBLN1	ITGB1	Fibro 5	Fibro 5
FBN1_ITGB1	FBN1	ITGB1	Fibro 5	Fibro 5
FN1_ITGB1	FN1	ITGB1	Fibro 5	Fibro 5
HSPG2_ITGB1	HSPG2	ITGB1	Fibro 5	Fibro 5
HSPG2_LRP1	HSPG2	LRP1	Fibro 5	Fibro 5
LAMA2_ITGB1	LAMA2	ITGB1	Fibro 5	Fibro 5
LAMC1_ITGB1	LAMC1	ITGB1	Fibro 5	Fibro 5
LGALS1_ITGB1	LGALS1	ITGB1	Fibro 5	Fibro 5
LUM_ITGB1	LUM	ITGB1	Fibro 5	Fibro 5
MFGE8_PDGFRB	MFGE8	PDGFRB	Fibro 5	Fibro 5
PSAP_LRP1	PSAP	LRP1	Fibro 5	Fibro 5
SERPINE2_LRP1	SERPINE2	LRP1	Fibro 5	Fibro 5
SERPING1_LRP1	SERPING1	LRP1	Fibro 5	Fibro 5
TFPI_LRP1	TFPI	LRP1	Fibro 5	Fibro 5
TIMP1_CD63	TIMP1	CD63	Fibro 5	Fibro 5
VCAN_ITGB1	VCAN	ITGB1	Fibro 5	Fibro 5
APP_LRP1	APP	LRP1	Fibro 5	Fibro 6
COL1A1_ITGB1	COL1A1	ITGB1	Fibro 5	Fibro 6
COL1A2_ITGB1	COL1A2	ITGB1	Fibro 5	Fibro 6
COL3A1_ITGB1	COL3A1	ITGB1	Fibro 5	Fibro 6
COL6A1_ITGB1	COL6A1	ITGB1	Fibro 5	Fibro 6
COL6A2_ITGB1	COL6A2	ITGB1	Fibro 5	Fibro 6
COL6A3_ITGB1	COL6A3	ITGB1	Fibro 5	Fibro 6
FBLN1_ITGB1	FBLN1	ITGB1	Fibro 5	Fibro 6
FBN1_ITGB1	FBN1	ITGB1	Fibro 5	Fibro 6
FN1_ITGB1	FN1	ITGB1	Fibro 5	Fibro 6
HSPG2_ITGB1	HSPG2	ITGB1	Fibro 5	Fibro 6
HSPG2_LRP1	HSPG2	LRP1	Fibro 5	Fibro 6
LAMA2_ITGB1	LAMA2	ITGB1	Fibro 5	Fibro 6
LAMC1_ITGB1	LAMC1	ITGB1	Fibro 5	Fibro 6
LGALS1_ITGB1	LGALS1	ITGB1	Fibro 5	Fibro 6
LUM_ITGB1	LUM	ITGB1	Fibro 5	Fibro 6
MFGE8_PDGFRB	MFGE8	PDGFRB	Fibro 5	Fibro 6
PSAP_LRP1	PSAP	LRP1	Fibro 5	Fibro 6
SERPINE2_LRP1	SERPINE2	LRP1	Fibro 5	Fibro 6
SERPING1_LRP1	SERPING1	LRP1	Fibro 5	Fibro 6
TFPI_LRP1	TFPI	LRP1	Fibro 5	Fibro 6
TIMP1_CD63	TIMP1	CD63	Fibro 5	Fibro 6
VCAN_ITGB1	VCAN	ITGB1	Fibro 5	Fibro 6

Non-obstructive				
Pair_Name	Ligand	Receptor	L_cell	R_cell
A2M_LRP1	A2M	LRP1	Fibro 5	Fibro 2
CALM1_RYR2	CALM1	RYR2	Fibro 5	Fibro 2
FN1_ITGB1	FN1	ITGB1	Fibro 5	Fibro 2
HSP90AA1_LRP1	HSP90AA1	LRP1	Fibro 5	Fibro 2
LPL_LRP1	LPL	LRP1	Fibro 5	Fibro 2
LUM_ITGB1	LUM	ITGB1	Fibro 5	Fibro 2
PSAP_LRP1	PSAP	LRP1	Fibro 5	Fibro 2
S100A1_RYR2	S100A1	RYR2	Fibro 5	Fibro 2
CALM1_RYR2	CALM1	RYR2	Fibro 5	Fibro 3
FN1_ITGB1	FN1	ITGB1	Fibro 5	Fibro 3
LUM_ITGB1	LUM	ITGB1	Fibro 5	Fibro 3
S100A1_RYR2	S100A1	RYR2	Fibro 5	Fibro 3
CALM1_RYR2	CALM1	RYR2	Fibro 5	Fibro 4
FN1_ITGB1	FN1	ITGB1	Fibro 5	Fibro 4
LUM_ITGB1	LUM	ITGB1	Fibro 5	Fibro 4
S100A1_RYR2	S100A1	RYR2	Fibro 5	Fibro 4
CALM1_RYR2	CALM1	RYR2	Fibro 5	Fibro 5
FN1_ITGB1	FN1	ITGB1	Fibro 5	Fibro 5
LUM_ITGB1	LUM	ITGB1	Fibro 5	Fibro 5
S100A1_RYR2	S100A1	RYR2	Fibro 5	Fibro 5
CALM1_RYR2	CALM1	RYR2	Fibro 5	Fibro 6
FN1_ITGB1	FN1	ITGB1	Fibro 5	Fibro 6
LUM_ITGB1	LUM	ITGB1	Fibro 5	Fibro 6
S100A1_RYR2	S100A1	RYR2	Fibro 5	Fibro 6

Supplemental Table 9. Fibroblast Communication with Fibroblasts and Cardiomyocyte Cluster 4

Normal				
**Pair_Name**	**Ligand**	**Receptor**	**L_cell**	**R_cell**
APP_LRP1	APP	LRP1	Fibro	Fibro
C3_CD81	C3	CD81	Fibro	Fibro
C3_LRP1	C3	LRP1	Fibro	Fibro
COL1A1_ITGB1	COL1A1	ITGB1	Fibro	Fibro
COL1A2_ITGB1	COL1A2	ITGB1	Fibro	Fibro
COL3A1_ITGB1	COL3A1	ITGB1	Fibro	Fibro
COL4A1_ITGB1	COL4A1	ITGB1	Fibro	Fibro
COL6A1_ITGB1	COL6A1	ITGB1	Fibro	Fibro
COL6A2_ITGB1	COL6A2	ITGB1	Fibro	Fibro
COL6A3_ITGB1	COL6A3	ITGB1	Fibro	Fibro
CTGF_LRP1	CTGF	LRP1	Fibro	Fibro
FBLN1_ITGB1	FBLN1	ITGB1	Fibro	Fibro
FBN1_ITGB1	FBN1	ITGB1	Fibro	Fibro
FN1_ITGB1	FN1	ITGB1	Fibro	Fibro
HSP90B1_LRP1	HSP90B1	LRP1	Fibro	Fibro
HSPG2_ITGB1	HSPG2	ITGB1	Fibro	Fibro
HSPG2_LRP1	HSPG2	LRP1	Fibro	Fibro
LAMA2_ITGB1	LAMA2	ITGB1	Fibro	Fibro
LAMB1_ITGB1	LAMB1	ITGB1	Fibro	Fibro
LAMC1_ITGB1	LAMC1	ITGB1	Fibro	Fibro
LGALS1_ITGB1	LGALS1	ITGB1	Fibro	Fibro
LUM_ITGB1	LUM	ITGB1	Fibro	Fibro
MFGE8_PDGFRB	MFGE8	PDGFRB	Fibro	Fibro
NID1_ITGB1	NID1	ITGB1	Fibro	Fibro
PSAP_LRP1	PSAP	LRP1	Fibro	Fibro
SERPINE2_LRP1	SERPINE2	LRP1	Fibro	Fibro
SERPING1_LRP1	SERPING1	LRP1	Fibro	Fibro
TFPI_LRP1	TFPI	LRP1	Fibro	Fibro
TIMP1_CD63	TIMP1	CD63	Fibro	Fibro
VCAN_ITGB1	VCAN	ITGB1	Fibro	Fibro
APP_CAV1	APP	CAV1	Fibro	CM 4
CALM2_CACNA1C	CALM2	CACNA1C	Fibro	CM 4
CALM2_INSR	CALM2	INSR	Fibro	CM 4
COL1A1_CD36	COL1A1	CD36	Fibro	CM 4
COL1A1_ITGB1	COL1A1	ITGB1	Fibro	CM 4
COL1A2_CD36	COL1A2	CD36	Fibro	CM 4
COL1A2_ITGB1	COL1A2	ITGB1	Fibro	CM 4
COL3A1_ITGB1	COL3A1	ITGB1	Fibro	CM 4
COL4A1_ITGB1	COL4A1	ITGB1	Fibro	CM 4
COL6A1_ITGB1	COL6A1	ITGB1	Fibro	CM 4
COL6A2_ITGB1	COL6A2	ITGB1	Fibro	CM 4
COL6A3_ITGB1	COL6A3	ITGB1	Fibro	CM 4
FBLN1_ITGB1	FBLN1	ITGB1	Fibro	CM 4
FBN1_ITGB1	FBN1	ITGB1	Fibro	CM 4
FN1_ITGB1	FN1	ITGB1	Fibro	CM 4
HSPG2_ITGB1	HSPG2	ITGB1	Fibro	CM 4
IGF1_INSR	IGF1	INSR	Fibro	CM 4
LAMA2_ITGB1	LAMA2	ITGB1	Fibro	CM 4
LAMB1_ITGB1	LAMB1	ITGB1	Fibro	CM 4
LAMC1_ITGB1	LAMC1	ITGB1	Fibro	CM 4
LGALS1_ITGB1	LGALS1	ITGB1	Fibro	CM 4
LUM_ITGB1	LUM	ITGB1	Fibro	CM 4
NID1_ITGB1	NID1	ITGB1	Fibro	CM 4
TIMP1_CD63	TIMP1	CD63	Fibro	CM 4
VCAN_ITGB1	VCAN	ITGB1	Fibro	CM 4

Non-obstructive				
**Pair_Name**	**Ligand**	**Receptor**	**L_cell**	**R_cell**
CALM1_RYR2	CALM1	RYR2	Fibro	Fibro
COL1A2_CD36	COL1A2	CD36	Fibro	Fibro
COL1A2_ITGB1	COL1A2	ITGB1	Fibro	Fibro
COL3A1_ITGB1	COL3A1	ITGB1	Fibro	Fibro
COL4A1_ITGB1	COL4A1	ITGB1	Fibro	Fibro
COL6A1_ITGB1	COL6A1	ITGB1	Fibro	Fibro
COL6A2_ITGB1	COL6A2	ITGB1	Fibro	Fibro
COL6A3_ITGB1	COL6A3	ITGB1	Fibro	Fibro
FN1_ITGB1	FN1	ITGB1	Fibro	Fibro
LAMA2_ITGB1	LAMA2	ITGB1	Fibro	Fibro
LGALS1_ITGB1	LGALS1	ITGB1	Fibro	Fibro
LUM_ITGB1	LUM	ITGB1	Fibro	Fibro
S100A1_RYR2	S100A1	RYR2	Fibro	Fibro
CALM1_RYR2	CALM1	RYR2	Fibro	CM 4
COL1A2_CD36	COL1A2	CD36	Fibro	CM 4
COL1A2_ITGB1	COL1A2	ITGB1	Fibro	CM 4
COL3A1_ITGB1	COL3A1	ITGB1	Fibro	CM 4
COL4A1_ITGB1	COL4A1	ITGB1	Fibro	CM 4
COL6A1_ITGB1	COL6A1	ITGB1	Fibro	CM 4
COL6A2_ITGB1	COL6A2	ITGB1	Fibro	CM 4
COL6A3_ITGB1	COL6A3	ITGB1	Fibro	CM 4
FN1_ITGB1	FN1	ITGB1	Fibro	CM 4
LAMA2_ITGB1	LAMA2	ITGB1	Fibro	CM 4
LGALS1_ITGB1	LGALS1	ITGB1	Fibro	CM 4
LUM_ITGB1	LUM	ITGB1	Fibro	CM 4
S100A1_RYR2	S100A1	RYR2	Fibro	CM 4

Supplemental Table 10. Endothelial Cell Communication to Fibroblasts, Cardiomyocyte Cluster 4 and Cardiomyocyte Cluster 8

Normal				
**Pair_Name**	**Ligand**	**Receptor**	**L_cell**	**R_cell**
A2M_LRP1	A2M	LRP1	EC	Fibro
APP_LRP1	APP	LRP1	EC	Fibro
COL1A1_ITGB1	COL1A1	ITGB1	EC	Fibro
COL1A2_ITGB1	COL1A2	ITGB1	EC	Fibro
COL3A1_ITGB1	COL3A1	ITGB1	EC	Fibro
COL6A1_ITGB1	COL6A1	ITGB1	EC	Fibro
COL6A2_ITGB1	COL6A2	ITGB1	EC	Fibro
FN1_ITGB1	FN1	ITGB1	EC	Fibro
GNAS_PTGIR	GNAS	PTGIR	EC	Fibro
HSP90AA1_LRP1	HSP90AA1	LRP1	EC	Fibro
HSP90B1_LRP1	HSP90B1	LRP1	EC	Fibro
HSPG2_ITGB1	HSPG2	ITGB1	EC	Fibro
HSPG2_LRP1	HSPG2	LRP1	EC	Fibro
LGALS1_ITGB1	LGALS1	ITGB1	EC	Fibro
LUM_ITGB1	LUM	ITGB1	EC	Fibro
MFGE8_PDGFRB	MFGE8	PDGFRB	EC	Fibro
PSAP_LRP1	PSAP	LRP1	EC	Fibro
TIMP1_CD63	TIMP1	CD63	EC	Fibro
VWF_LRP1	VWF	LRP1	EC	Fibro
APP_CAV1	APP	CAV1	EC	CM 4
CALM1_CACNA1C	CALM1	CACNA1C	EC	CM 4
CALM1_INSR	CALM1	INSR	EC	CM 4
CALM1_RYR2	CALM1	RYR2	EC	CM 4
CALM2_CACNA1C	CALM2	CACNA1C	EC	CM 4
CALM2_INSR	CALM2	INSR	EC	CM 4
COL1A1_CD36	COL1A1	CD36	EC	CM 4
COL1A1_ITGB1	COL1A1	ITGB1	EC	CM 4
COL1A2_CD36	COL1A2	CD36	EC	CM 4
COL1A2_ITGB1	COL1A2	ITGB1	EC	CM 4
COL3A1_ITGB1	COL3A1	ITGB1	EC	CM 4
COL6A1_ITGB1	COL6A1	ITGB1	EC	CM 4
COL6A2_ITGB1	COL6A2	ITGB1	EC	CM 4
FN1_ITGB1	FN1	ITGB1	EC	CM 4
HSPG2_ITGB1	HSPG2	ITGB1	EC	CM 4
LGALS1_ITGB1	LGALS1	ITGB1	EC	CM 4
LUM_ITGB1	LUM	ITGB1	EC	CM 4
TIMP1_CD63	TIMP1	CD63	EC	CM 4
CALM1_CACNA1C	CALM1	CACNA1C	EC	CM 8
CALM1_INSR	CALM1	INSR	EC	CM 8
CALM1_RYR2	CALM1	RYR2	EC	CM 8
CALM2_CACNA1C	CALM2	CACNA1C	EC	CM 8
CALM2_INSR	CALM2	INSR	EC	CM 8
COL1A1_CD36	COL1A1	CD36	EC	CM 8
COL1A1_ITGB1	COL1A1	ITGB1	EC	CM 8
COL1A2_CD36	COL1A2	CD36	EC	CM 8
COL1A2_ITGB1	COL1A2	ITGB1	EC	CM 8
COL3A1_ITGB1	COL3A1	ITGB1	EC	CM 8
COL6A1_ITGB1	COL6A1	ITGB1	EC	CM 8
COL6A2_ITGB1	COL6A2	ITGB1	EC	CM 8
FN1_ITGB1	FN1	ITGB1	EC	CM 8
HSPG2_ITGB1	HSPG2	ITGB1	EC	CM 8
LGALS1_ITGB1	LGALS1	ITGB1	EC	CM 8
LUM_ITGB1	LUM	ITGB1	EC	CM 8
TIMP1_CD63	TIMP1	CD63	EC	CM 8

Non-obstructive				
**Pair_Name**	**Ligand**	**Receptor**	**L_cell**	**R_cell**
CALM1_RYR2	CALM1	RYR2	EC	Fibro
FN1_ITGB1	FN1	ITGB1	EC	Fibro
LGALS1_ITGB1	LGALS1	ITGB1	EC	Fibro
S100A1_RYR2	S100A1	RYR2	EC	Fibro
CALM1_RYR2	CALM1	RYR2	EC	CM 4
FN1_ITGB1	FN1	ITGB1	EC	CM 4
LGALS1_ITGB1	LGALS1	ITGB1	EC	CM 4
S100A1_RYR2	S100A1	RYR2	EC	CM 4
CALM1_CACNA1C	CALM1	CACNA1C	EC	CM 8
CALM1_RYR2	CALM1	RYR2	EC	CM 8
FN1_ITGB1	FN1	ITGB1	EC	CM 8
LGALS1_ITGB1	LGALS1	ITGB1	EC	CM 8
S100A1_RYR2	S100A1	RYR2	EC	CM 8

Supplemental Table 11. Cardiomyocyte Cluster 2, 5, Fibroblast and Smooth Muscle Cell Communication to Cardiomyocyte Cluster 9

Normal				
**Pair_Name**	**Ligand**	**Receptor**	**L_cell**	**R_cell**
COL1A2_CD36	COL1A2	CD36	CM 2	CM 9
TIMP1_CD63	TIMP1	CD63	CM 2	CM 9
CALM1_RYR2	CALM1	RYR2	CM 5	CM 9
COL1A2_CD36	COL1A2	CD36	CM 5	CM 9
S100A1_RYR2	S100A1	RYR2	CM 5	CM 9
TIMP1_CD63	TIMP1	CD63	CM 5	CM 9
COL1A1_CD36	COL1A1	CD36	Fibro	CM 9
COL1A2_CD36	COL1A2	CD36	Fibro	CM 9
TIMP1_CD63	TIMP1	CD63	Fibro	CM 9
COL1A2_CD36	COL1A2	CD36	SMC	CM 9
TIMP1_CD63	TIMP1	CD63	SMC	CM 9

Non-obstructive				
**Pair_Name**	**Ligand**	**Receptor**	**L_cell**	**R_cell**
CALM1_CACNA1C	CALM1	CACNA1C	CM 2	CM 9
CALM1_PDE1C	CALM1	PDE1C	CM 2	CM 9
CALM1_RYR2	CALM1	RYR2	CM 2	CM 9
CALM2_CACNA1C	CALM2	CACNA1C	CM 2	CM 9
CALM2_PDE1C	CALM2	PDE1C	CM 2	CM 9
COL1A2_CD36	COL1A2	CD36	CM 2	CM 9
COL1A2_ITGB1	COL1A2	ITGB1	CM 2	CM 9
COL6A2_ITGB1	COL6A2	ITGB1	CM 2	CM 9
FN1_ITGB1	FN1	ITGB1	CM 2	CM 9
LGALS1_ITGB1	LGALS1	ITGB1	CM 2	CM 9
LUM_ITGB1	LUM	ITGB1	CM 2	CM 9
S100A1_RYR2	S100A1	RYR2	CM 2	CM 9
TIMP1_CD63	TIMP1	CD63	CM 2	CM 9
CALM1_CACNA1C	CALM1	CACNA1C	CM 5	CM 9
CALM1_PDE1C	CALM1	PDE1C	CM 5	CM 9
CALM1_RYR2	CALM1	RYR2	CM 5	CM 9
CALM2_CACNA1C	CALM2	CACNA1C	CM 5	CM 9
CALM2_PDE1C	CALM2	PDE1C	CM 5	CM 9
CALM3_CACNA1C	CALM3	CACNA1C	CM 5	CM 9
CALM3_PDE1C	CALM3	PDE1C	CM 5	CM 9
CALM3_RYR2	CALM3	RYR2	CM 5	CM 9
COL1A2_CD36	COL1A2	CD36	CM 5	CM 9
COL1A2_ITGB1	COL1A2	ITGB1	CM 5	CM 9
COL6A1_ITGB1	COL6A1	ITGB1	CM 5	CM 9
COL6A2_ITGB1	COL6A2	ITGB1	CM 5	CM 9
FN1_ITGB1	FN1	ITGB1	CM 5	CM 9
LAMB2_ITGB1	LAMB2	ITGB1	CM 5	CM 9
LGALS1_ITGB1	LGALS1	ITGB1	CM 5	CM 9
LGALS3BP_ITGB1	LGALS3BP	ITGB1	CM 5	CM 9
LUM_ITGB1	LUM	ITGB1	CM 5	CM 9
S100A1_RYR2	S100A1	RYR2	CM 5	CM 9
TGM2_ITGB1	TGM2	ITGB1	CM 5	CM 9
TIMP1_CD63	TIMP1	CD63	CM 5	CM 9
CALM1_CACNA1C	CALM1	CACNA1C	Fibro	CM 9
CALM1_PDE1C	CALM1	PDE1C	Fibro	CM 9
CALM1_RYR2	CALM1	RYR2	Fibro	CM 9
COL1A2_CD36	COL1A2	CD36	Fibro	CM 9
COL1A2_ITGB1	COL1A2	ITGB1	Fibro	CM 9
COL3A1_ITGB1	COL3A1	ITGB1	Fibro	CM 9
COL4A1_ITGB1	COL4A1	ITGB1	Fibro	CM 9
COL6A1_ITGB1	COL6A1	ITGB1	Fibro	CM 9
COL6A2_ITGB1	COL6A2	ITGB1	Fibro	CM 9
COL6A3_ITGB1	COL6A3	ITGB1	Fibro	CM 9
FN1_ITGB1	FN1	ITGB1	Fibro	CM 9
LAMA2_ITGB1	LAMA2	ITGB1	Fibro	CM 9
LGALS1_ITGB1	LGALS1	ITGB1	Fibro	CM 9
LUM_ITGB1	LUM	ITGB1	Fibro	CM 9
S100A1_RYR2	S100A1	RYR2	Fibro	CM 9
CALM1_CACNA1C	CALM1	CACNA1C	SMC	CM 9
CALM1_PDE1C	CALM1	PDE1C	SMC	CM 9
CALM1_RYR2	CALM1	RYR2	SMC	CM 9
CALM2_CACNA1C	CALM2	CACNA1C	SMC	CM 9
CALM2_PDE1C	CALM2	PDE1C	SMC	CM 9
COL1A2_CD36	COL1A2	CD36	SMC	CM 9
COL1A2_ITGB1	COL1A2	ITGB1	SMC	CM 9
COL4A1_ITGB1	COL4A1	ITGB1	SMC	CM 9
COL6A1_ITGB1	COL6A1	ITGB1	SMC	CM 9
COL6A2_ITGB1	COL6A2	ITGB1	SMC	CM 9
FN1_ITGB1	FN1	ITGB1	SMC	CM 9
LGALS1_ITGB1	LGALS1	ITGB1	SMC	CM 9
S100A1_RYR2	S100A1	RYR2	SMC	CM 9

Supplemental Table 12. Cardiomyocyte Cluster 5 Communication to Dendritic Cells

Normal				
**Pair_Name**	**Ligand**	**Receptor**	**L_cell**	**R_cell**
COL1A2_CD36	COL1A2	CD36	CM 5	DC
LGALS1_PTPRC	LGALS1	PTPRC	CM 5	DC
TIMP1_CD63	TIMP1	CD63	CM 5	DC

Non-obstructive				
**Pair_Name**	**Ligand**	**Receptor**	**L_cell**	**R_cell**
CALM1_RYR2	CALM1	RYR2	CM 5	DC
CALM3_RYR2	CALM3	RYR2	CM 5	DC
COL1A2_CD36	COL1A2	CD36	CM 5	DC
COL1A2_ITGB1	COL1A2	ITGB1	CM 5	DC
COL6A1_ITGB1	COL6A1	ITGB1	CM 5	DC
COL6A2_ITGB1	COL6A2	ITGB1	CM 5	DC
FN1_ITGB1	FN1	ITGB1	CM 5	DC
LAMB2_ITGB1	LAMB2	ITGB1	CM 5	DC
LGALS1_ITGB1	LGALS1	ITGB1	CM 5	DC
LGALS3BP_ITGB1	LGALS3BP	ITGB1	CM 5	DC
LUM_ITGB1	LUM	ITGB1	CM 5	DC
MIF_CD74	MIF	CD74	CM 5	DC
S100A1_RYR2	S100A1	RYR2	CM 5	DC
TGM2_ITGB1	TGM2	ITGB1	CM 5	DC
TIMP1_CD63	TIMP1	CD63	CM 5	DC

Supplemental Table 13. Fibroblast Cluster 2 and 5 Communication to Fibroblast Clusters 2-6.

Normal				
Pair_Name	Ligand	Receptor	L_cell	R_cell
APP_LRP1	APP	LRP1	Fibro 2	Fibro 2
C3_CD81	C3	CD81	Fibro 2	Fibro 2
C3_LRP1	C3	LRP1	Fibro 2	Fibro 2
CALR_LRP1	CALR	LRP1	Fibro 2	Fibro 2
CD99_CD81	CD99	CD81	Fibro 2	Fibro 2
COL18A1_ITGB1	COL18A1	ITGB1	Fibro 2	Fibro 2
COL1A1_ITGB1	COL1A1	ITGB1	Fibro 2	Fibro 2
COL1A2_ITGB1	COL1A2	ITGB1	Fibro 2	Fibro 2
COL3A1_ITGB1	COL3A1	ITGB1	Fibro 2	Fibro 2
COL4A1_ITGB1	COL4A1	ITGB1	Fibro 2	Fibro 2
COL5A1_ITGB1	COL5A1	ITGB1	Fibro 2	Fibro 2
COL5A2_ITGB1	COL5A2	ITGB1	Fibro 2	Fibro 2
COL6A1_ITGB1	COL6A1	ITGB1	Fibro 2	Fibro 2
COL6A2_ITGB1	COL6A2	ITGB1	Fibro 2	Fibro 2
COL6A3_ITGB1	COL6A3	ITGB1	Fibro 2	Fibro 2
CTGF_LRP1	CTGF	LRP1	Fibro 2	Fibro 2
FBLN1_ITGB1	FBLN1	ITGB1	Fibro 2	Fibro 2
FBN1_ITGB1	FBN1	ITGB1	Fibro 2	Fibro 2
FN1_ITGB1	FN1	ITGB1	Fibro 2	Fibro 2
FN1_SDC2	FN1	SDC2	Fibro 2	Fibro 2
HLA-A_APLP2	HLA-A	APLP2	Fibro 2	Fibro 2
HSP90AA1_LRP1	HSP90AA1	LRP1	Fibro 2	Fibro 2
HSP90B1_LRP1	HSP90B1	LRP1	Fibro 2	Fibro 2
HSPG2_ITGB1	HSPG2	ITGB1	Fibro 2	Fibro 2
HSPG2_LRP1	HSPG2	LRP1	Fibro 2	Fibro 2
LAMA2_ITGB1	LAMA2	ITGB1	Fibro 2	Fibro 2
LAMB1_ITGB1	LAMB1	ITGB1	Fibro 2	Fibro 2
LAMC1_ITGB1	LAMC1	ITGB1	Fibro 2	Fibro 2
LGALS1_ITGB1	LGALS1	ITGB1	Fibro 2	Fibro 2
LGALS3BP_ITGB1	LGALS3BP	ITGB1	Fibro 2	Fibro 2
LRPAP1_LRP1	LRPAP1	LRP1	Fibro 2	Fibro 2
LUM_ITGB1	LUM	ITGB1	Fibro 2	Fibro 2
MFGE8_PDGFRB	MFGE8	PDGFRB	Fibro 2	Fibro 2
MMP2_SDC2	MMP2	SDC2	Fibro 2	Fibro 2
NID1_ITGB1	NID1	ITGB1	Fibro 2	Fibro 2
PSAP_LRP1	PSAP	LRP1	Fibro 2	Fibro 2
SERPINE2_LRP1	SERPINE2	LRP1	Fibro 2	Fibro 2
SERPING1_LRP1	SERPING1	LRP1	Fibro 2	Fibro 2
TFPI_LRP1	TFPI	LRP1	Fibro 2	Fibro 2
THBS2_ITGB1	THBS2	ITGB1	Fibro 2	Fibro 2
TIMP1_CD63	TIMP1	CD63	Fibro 2	Fibro 2
TIMP2_ITGB1	TIMP2	ITGB1	Fibro 2	Fibro 2
VCAN_ITGB1	VCAN	ITGB1	Fibro 2	Fibro 2
APP_LRP1	APP	LRP1	Fibro 2	Fibro 3
C3_CD81	C3	CD81	Fibro 2	Fibro 3
C3_LRP1	C3	LRP1	Fibro 2	Fibro 3
CALR_LRP1	CALR	LRP1	Fibro 2	Fibro 3
CD99_CD81	CD99	CD81	Fibro 2	Fibro 3
COL18A1_ITGB1	COL18A1	ITGB1	Fibro 2	Fibro 3
COL1A1_ITGB1	COL1A1	ITGB1	Fibro 2	Fibro 3
COL1A2_ITGB1	COL1A2	ITGB1	Fibro 2	Fibro 3
COL3A1_ITGB1	COL3A1	ITGB1	Fibro 2	Fibro 3
COL4A1_ITGB1	COL4A1	ITGB1	Fibro 2	Fibro 3
COL5A1_ITGB1	COL5A1	ITGB1	Fibro 2	Fibro 3
COL5A2_ITGB1	COL5A2	ITGB1	Fibro 2	Fibro 3
COL6A1_ITGB1	COL6A1	ITGB1	Fibro 2	Fibro 3
COL6A2_ITGB1	COL6A2	ITGB1	Fibro 2	Fibro 3
COL6A3_ITGB1	COL6A3	ITGB1	Fibro 2	Fibro 3
CTGF_LRP1	CTGF	LRP1	Fibro 2	Fibro 3
FBLN1_ITGB1	FBLN1	ITGB1	Fibro 2	Fibro 3
FBN1_ITGB1	FBN1	ITGB1	Fibro 2	Fibro 3
FN1_ITGB1	FN1	ITGB1	Fibro 2	Fibro 3
HSP90AA1_LRP1	HSP90AA1	LRP1	Fibro 2	Fibro 3
HSP90B1_LRP1	HSP90B1	LRP1	Fibro 2	Fibro 3
HSPG2_ITGB1	HSPG2	ITGB1	Fibro 2	Fibro 3
HSPG2_LRP1	HSPG2	LRP1	Fibro 2	Fibro 3
LAMA2_ITGB1	LAMA2	ITGB1	Fibro 2	Fibro 3
LAMB1_ITGB1	LAMB1	ITGB1	Fibro 2	Fibro 3
LAMC1_ITGB1	LAMC1	ITGB1	Fibro 2	Fibro 3
LGALS1_ITGB1	LGALS1	ITGB1	Fibro 2	Fibro 3
LGALS3BP_ITGB1	LGALS3BP	ITGB1	Fibro 2	Fibro 3
LRPAP1_LRP1	LRPAP1	LRP1	Fibro 2	Fibro 3
LUM_ITGB1	LUM	ITGB1	Fibro 2	Fibro 3
MFGE8_PDGFRB	MFGE8	PDGFRB	Fibro 2	Fibro 3
NID1_ITGB1	NID1	ITGB1	Fibro 2	Fibro 3
PSAP_LRP1	PSAP	LRP1	Fibro 2	Fibro 3
SERPINE2_LRP1	SERPINE2	LRP1	Fibro 2	Fibro 3
SERPING1_LRP1	SERPING1	LRP1	Fibro 2	Fibro 3
TFPI_LRP1	TFPI	LRP1	Fibro 2	Fibro 3
THBS2_ITGB1	THBS2	ITGB1	Fibro 2	Fibro 3
TIMP1_CD63	TIMP1	CD63	Fibro 2	Fibro 3
TIMP2_ITGB1	TIMP2	ITGB1	Fibro 2	Fibro 3
VCAN_ITGB1	VCAN	ITGB1	Fibro 2	Fibro 3
APP_LRP1	APP	LRP1	Fibro 2	Fibro 4
C3_CD81	C3	CD81	Fibro 2	Fibro 4
C3_LRP1	C3	LRP1	Fibro 2	Fibro 4
CALR_LRP1	CALR	LRP1	Fibro 2	Fibro 4
CD99_CD81	CD99	CD81	Fibro 2	Fibro 4
COL18A1_ITGB1	COL18A1	ITGB1	Fibro 2	Fibro 4
COL1A1_ITGB1	COL1A1	ITGB1	Fibro 2	Fibro 4
COL1A2_ITGB1	COL1A2	ITGB1	Fibro 2	Fibro 4
COL3A1_ITGB1	COL3A1	ITGB1	Fibro 2	Fibro 4
COL4A1_ITGB1	COL4A1	ITGB1	Fibro 2	Fibro 4
COL5A1_ITGB1	COL5A1	ITGB1	Fibro 2	Fibro 4
COL5A2_ITGB1	COL5A2	ITGB1	Fibro 2	Fibro 4
COL6A1_ITGB1	COL6A1	ITGB1	Fibro 2	Fibro 4
COL6A2_ITGB1	COL6A2	ITGB1	Fibro 2	Fibro 4
COL6A3_ITGB1	COL6A3	ITGB1	Fibro 2	Fibro 4
CTGF_LRP1	CTGF	LRP1	Fibro 2	Fibro 4
FBLN1_ITGB1	FBLN1	ITGB1	Fibro 2	Fibro 4
FBN1_ITGB1	FBN1	ITGB1	Fibro 2	Fibro 4
FN1_ITGB1	FN1	ITGB1	Fibro 2	Fibro 4
HSP90AA1_LRP1	HSP90AA1	LRP1	Fibro 2	Fibro 4
HSP90B1_LRP1	HSP90B1	LRP1	Fibro 2	Fibro 4
HSPG2_ITGB1	HSPG2	ITGB1	Fibro 2	Fibro 4
HSPG2_LRP1	HSPG2	LRP1	Fibro 2	Fibro 4
LAMA2_ITGB1	LAMA2	ITGB1	Fibro 2	Fibro 4
LAMB1_ITGB1	LAMB1	ITGB1	Fibro 2	Fibro 4
LAMC1_ITGB1	LAMC1	ITGB1	Fibro 2	Fibro 4
LGALS1_ITGB1	LGALS1	ITGB1	Fibro 2	Fibro 4
LGALS3BP_ITGB1	LGALS3BP	ITGB1	Fibro 2	Fibro 4
LRPAP1_LRP1	LRPAP1	LRP1	Fibro 2	Fibro 4
LUM_ITGB1	LUM	ITGB1	Fibro 2	Fibro 4
MFGE8_PDGFRB	MFGE8	PDGFRB	Fibro 2	Fibro 4
NID1_ITGB1	NID1	ITGB1	Fibro 2	Fibro 4
PSAP_LRP1	PSAP	LRP1	Fibro 2	Fibro 4
SERPINE2_LRP1	SERPINE2	LRP1	Fibro 2	Fibro 4
SERPING1_LRP1	SERPING1	LRP1	Fibro 2	Fibro 4
TFPI_LRP1	TFPI	LRP1	Fibro 2	Fibro 4
THBS2_ITGB1	THBS2	ITGB1	Fibro 2	Fibro 4
TIMP1_CD63	TIMP1	CD63	Fibro 2	Fibro 4
TIMP2_ITGB1	TIMP2	ITGB1	Fibro 2	Fibro 4
VCAN_ITGB1	VCAN	ITGB1	Fibro 2	Fibro 4
APP_LRP1	APP	LRP1	Fibro 2	Fibro 5
C3_CD81	C3	CD81	Fibro 2	Fibro 5
C3_LRP1	C3	LRP1	Fibro 2	Fibro 5
CALR_LRP1	CALR	LRP1	Fibro 2	Fibro 5
CD99_CD81	CD99	CD81	Fibro 2	Fibro 5
COL18A1_ITGB1	COL18A1	ITGB1	Fibro 2	Fibro 5
COL1A1_CD36	COL1A1	CD36	Fibro 2	Fibro 5
COL1A1_ITGB1	COL1A1	ITGB1	Fibro 2	Fibro 5
COL1A2_CD36	COL1A2	CD36	Fibro 2	Fibro 5
COL1A2_ITGB1	COL1A2	ITGB1	Fibro 2	Fibro 5
COL3A1_ITGB1	COL3A1	ITGB1	Fibro 2	Fibro 5
COL4A1_ITGB1	COL4A1	ITGB1	Fibro 2	Fibro 5
COL5A1_ITGB1	COL5A1	ITGB1	Fibro 2	Fibro 5
COL5A2_ITGB1	COL5A2	ITGB1	Fibro 2	Fibro 5
COL6A1_ITGB1	COL6A1	ITGB1	Fibro 2	Fibro 5
COL6A2_ITGB1	COL6A2	ITGB1	Fibro 2	Fibro 5
COL6A3_ITGB1	COL6A3	ITGB1	Fibro 2	Fibro 5
CTGF_LRP1	CTGF	LRP1	Fibro 2	Fibro 5
FBLN1_ITGB1	FBLN1	ITGB1	Fibro 2	Fibro 5
FBN1_ITGB1	FBN1	ITGB1	Fibro 2	Fibro 5
FN1_ITGB1	FN1	ITGB1	Fibro 2	Fibro 5
HSP90AA1_LRP1	HSP90AA1	LRP1	Fibro 2	Fibro 5
HSP90B1_LRP1	HSP90B1	LRP1	Fibro 2	Fibro 5
HSPG2_ITGB1	HSPG2	ITGB1	Fibro 2	Fibro 5
HSPG2_LRP1	HSPG2	LRP1	Fibro 2	Fibro 5
LAMA2_ITGB1	LAMA2	ITGB1	Fibro 2	Fibro 5
LAMB1_ITGB1	LAMB1	ITGB1	Fibro 2	Fibro 5
LAMC1_ITGB1	LAMC1	ITGB1	Fibro 2	Fibro 5
LGALS1_ITGB1	LGALS1	ITGB1	Fibro 2	Fibro 5
LGALS3BP_ITGB1	LGALS3BP	ITGB1	Fibro 2	Fibro 5
LRPAP1_LRP1	LRPAP1	LRP1	Fibro 2	Fibro 5
LUM_ITGB1	LUM	ITGB1	Fibro 2	Fibro 5
MFGE8_PDGFRB	MFGE8	PDGFRB	Fibro 2	Fibro 5
NID1_ITGB1	NID1	ITGB1	Fibro 2	Fibro 5
PSAP_LRP1	PSAP	LRP1	Fibro 2	Fibro 5
SERPINE2_LRP1	SERPINE2	LRP1	Fibro 2	Fibro 5
SERPING1_LRP1	SERPING1	LRP1	Fibro 2	Fibro 5
TFPI_LRP1	TFPI	LRP1	Fibro 2	Fibro 5
THBS2_CD36	THBS2	CD36	Fibro 2	Fibro 5
THBS2_ITGB1	THBS2	ITGB1	Fibro 2	Fibro 5
TIMP1_CD63	TIMP1	CD63	Fibro 2	Fibro 5
TIMP2_ITGB1	TIMP2	ITGB1	Fibro 2	Fibro 5
VCAN_ITGB1	VCAN	ITGB1	Fibro 2	Fibro 5
APP_LRP1	APP	LRP1	Fibro 2	Fibro 6
C3_CD81	C3	CD81	Fibro 2	Fibro 6
C3_LRP1	C3	LRP1	Fibro 2	Fibro 6
CALR_LRP1	CALR	LRP1	Fibro 2	Fibro 6
CD99_CD81	CD99	CD81	Fibro 2	Fibro 6
COL18A1_ITGB1	COL18A1	ITGB1	Fibro 2	Fibro 6
COL1A1_ITGB1	COL1A1	ITGB1	Fibro 2	Fibro 6
COL1A2_ITGB1	COL1A2	ITGB1	Fibro 2	Fibro 6
COL3A1_ITGB1	COL3A1	ITGB1	Fibro 2	Fibro 6
COL4A1_ITGB1	COL4A1	ITGB1	Fibro 2	Fibro 6
COL5A1_ITGB1	COL5A1	ITGB1	Fibro 2	Fibro 6
COL5A2_ITGB1	COL5A2	ITGB1	Fibro 2	Fibro 6
COL6A1_ITGB1	COL6A1	ITGB1	Fibro 2	Fibro 6
COL6A2_ITGB1	COL6A2	ITGB1	Fibro 2	Fibro 6
COL6A3_ITGB1	COL6A3	ITGB1	Fibro 2	Fibro 6
CTGF_LRP1	CTGF	LRP1	Fibro 2	Fibro 6
FBLN1_ITGB1	FBLN1	ITGB1	Fibro 2	Fibro 6
FBN1_ITGB1	FBN1	ITGB1	Fibro 2	Fibro 6
FN1_ITGB1	FN1	ITGB1	Fibro 2	Fibro 6
HSP90AA1_LRP1	HSP90AA1	LRP1	Fibro 2	Fibro 6
HSP90B1_LRP1	HSP90B1	LRP1	Fibro 2	Fibro 6
HSPG2_ITGB1	HSPG2	ITGB1	Fibro 2	Fibro 6
HSPG2_LRP1	HSPG2	LRP1	Fibro 2	Fibro 6
LAMA2_ITGB1	LAMA2	ITGB1	Fibro 2	Fibro 6
LAMB1_ITGB1	LAMB1	ITGB1	Fibro 2	Fibro 6
LAMC1_ITGB1	LAMC1	ITGB1	Fibro 2	Fibro 6
LGALS1_ITGB1	LGALS1	ITGB1	Fibro 2	Fibro 6
LGALS3BP_ITGB1	LGALS3BP	ITGB1	Fibro 2	Fibro 6
LRPAP1_LRP1	LRPAP1	LRP1	Fibro 2	Fibro 6
LUM_ITGB1	LUM	ITGB1	Fibro 2	Fibro 6
MFGE8_PDGFRB	MFGE8	PDGFRB	Fibro 2	Fibro 6
NID1_ITGB1	NID1	ITGB1	Fibro 2	Fibro 6
PSAP_LRP1	PSAP	LRP1	Fibro 2	Fibro 6
SERPINE2_LRP1	SERPINE2	LRP1	Fibro 2	Fibro 6
SERPING1_LRP1	SERPING1	LRP1	Fibro 2	Fibro 6
TFPI_LRP1	TFPI	LRP1	Fibro 2	Fibro 6
THBS2_ITGB1	THBS2	ITGB1	Fibro 2	Fibro 6
TIMP1_CD63	TIMP1	CD63	Fibro 2	Fibro 6
TIMP2_ITGB1	TIMP2	ITGB1	Fibro 2	Fibro 6
VCAN_ITGB1	VCAN	ITGB1	Fibro 2	Fibro 6
APP_LRP1	APP	LRP1	Fibro 5	Fibro 2
COL1A1_ITGB1	COL1A1	ITGB1	Fibro 5	Fibro 2
COL1A2_ITGB1	COL1A2	ITGB1	Fibro 5	Fibro 2
COL3A1_ITGB1	COL3A1	ITGB1	Fibro 5	Fibro 2
COL6A1_ITGB1	COL6A1	ITGB1	Fibro 5	Fibro 2
COL6A2_ITGB1	COL6A2	ITGB1	Fibro 5	Fibro 2
COL6A3_ITGB1	COL6A3	ITGB1	Fibro 5	Fibro 2
FBLN1_ITGB1	FBLN1	ITGB1	Fibro 5	Fibro 2
FBN1_ITGB1	FBN1	ITGB1	Fibro 5	Fibro 2
FN1_ITGB1	FN1	ITGB1	Fibro 5	Fibro 2
FN1_SDC2	FN1	SDC2	Fibro 5	Fibro 2
HLA-A_APLP2	HLA-A	APLP2	Fibro 5	Fibro 2
HSPG2_ITGB1	HSPG2	ITGB1	Fibro 5	Fibro 2
HSPG2_LRP1	HSPG2	LRP1	Fibro 5	Fibro 2
LAMA2_ITGB1	LAMA2	ITGB1	Fibro 5	Fibro 2
LAMC1_ITGB1	LAMC1	ITGB1	Fibro 5	Fibro 2
LGALS1_ITGB1	LGALS1	ITGB1	Fibro 5	Fibro 2
LUM_ITGB1	LUM	ITGB1	Fibro 5	Fibro 2
MFGE8_PDGFRB	MFGE8	PDGFRB	Fibro 5	Fibro 2
MMP2_SDC2	MMP2	SDC2	Fibro 5	Fibro 2
PSAP_LRP1	PSAP	LRP1	Fibro 5	Fibro 2
SERPINE2_LRP1	SERPINE2	LRP1	Fibro 5	Fibro 2
SERPING1_LRP1	SERPING1	LRP1	Fibro 5	Fibro 2
TFPI_LRP1	TFPI	LRP1	Fibro 5	Fibro 2
TIMP1_CD63	TIMP1	CD63	Fibro 5	Fibro 2
VCAN_ITGB1	VCAN	ITGB1	Fibro 5	Fibro 2
APP_LRP1	APP	LRP1	Fibro 5	Fibro 3
COL1A1_ITGB1	COL1A1	ITGB1	Fibro 5	Fibro 3
COL1A2_ITGB1	COL1A2	ITGB1	Fibro 5	Fibro 3
COL3A1_ITGB1	COL3A1	ITGB1	Fibro 5	Fibro 3
COL6A1_ITGB1	COL6A1	ITGB1	Fibro 5	Fibro 3
COL6A2_ITGB1	COL6A2	ITGB1	Fibro 5	Fibro 3
COL6A3_ITGB1	COL6A3	ITGB1	Fibro 5	Fibro 3
FBLN1_ITGB1	FBLN1	ITGB1	Fibro 5	Fibro 3
FBN1_ITGB1	FBN1	ITGB1	Fibro 5	Fibro 3
FN1_ITGB1	FN1	ITGB1	Fibro 5	Fibro 3
HSPG2_ITGB1	HSPG2	ITGB1	Fibro 5	Fibro 3
HSPG2_LRP1	HSPG2	LRP1	Fibro 5	Fibro 3
LAMA2_ITGB1	LAMA2	ITGB1	Fibro 5	Fibro 3
LAMC1_ITGB1	LAMC1	ITGB1	Fibro 5	Fibro 3
LGALS1_ITGB1	LGALS1	ITGB1	Fibro 5	Fibro 3
LUM_ITGB1	LUM	ITGB1	Fibro 5	Fibro 3
MFGE8_PDGFRB	MFGE8	PDGFRB	Fibro 5	Fibro 3
PSAP_LRP1	PSAP	LRP1	Fibro 5	Fibro 3
SERPINE2_LRP1	SERPINE2	LRP1	Fibro 5	Fibro 3
SERPING1_LRP1	SERPING1	LRP1	Fibro 5	Fibro 3
TFPI_LRP1	TFPI	LRP1	Fibro 5	Fibro 3
TIMP1_CD63	TIMP1	CD63	Fibro 5	Fibro 3
VCAN_ITGB1	VCAN	ITGB1	Fibro 5	Fibro 3
APP_LRP1	APP	LRP1	Fibro 5	Fibro 4
COL1A1_ITGB1	COL1A1	ITGB1	Fibro 5	Fibro 4
COL1A2_ITGB1	COL1A2	ITGB1	Fibro 5	Fibro 4
COL3A1_ITGB1	COL3A1	ITGB1	Fibro 5	Fibro 4
COL6A1_ITGB1	COL6A1	ITGB1	Fibro 5	Fibro 4
COL6A2_ITGB1	COL6A2	ITGB1	Fibro 5	Fibro 4
COL6A3_ITGB1	COL6A3	ITGB1	Fibro 5	Fibro 4
FBLN1_ITGB1	FBLN1	ITGB1	Fibro 5	Fibro 4
FBN1_ITGB1	FBN1	ITGB1	Fibro 5	Fibro 4
FN1_ITGB1	FN1	ITGB1	Fibro 5	Fibro 4
HSPG2_ITGB1	HSPG2	ITGB1	Fibro 5	Fibro 4
HSPG2_LRP1	HSPG2	LRP1	Fibro 5	Fibro 4
LAMA2_ITGB1	LAMA2	ITGB1	Fibro 5	Fibro 4
LAMC1_ITGB1	LAMC1	ITGB1	Fibro 5	Fibro 4
LGALS1_ITGB1	LGALS1	ITGB1	Fibro 5	Fibro 4
LUM_ITGB1	LUM	ITGB1	Fibro 5	Fibro 4
MFGE8_PDGFRB	MFGE8	PDGFRB	Fibro 5	Fibro 4
PSAP_LRP1	PSAP	LRP1	Fibro 5	Fibro 4
SERPINE2_LRP1	SERPINE2	LRP1	Fibro 5	Fibro 4
SERPING1_LRP1	SERPING1	LRP1	Fibro 5	Fibro 4
TFPI_LRP1	TFPI	LRP1	Fibro 5	Fibro 4
TIMP1_CD63	TIMP1	CD63	Fibro 5	Fibro 4
VCAN_ITGB1	VCAN	ITGB1	Fibro 5	Fibro 4
APP_LRP1	APP	LRP1	Fibro 5	Fibro 5
COL1A1_CD36	COL1A1	CD36	Fibro 5	Fibro 5
COL1A1_ITGB1	COL1A1	ITGB1	Fibro 5	Fibro 5
COL1A2_CD36	COL1A2	CD36	Fibro 5	Fibro 5
COL1A2_ITGB1	COL1A2	ITGB1	Fibro 5	Fibro 5
COL3A1_ITGB1	COL3A1	ITGB1	Fibro 5	Fibro 5
COL6A1_ITGB1	COL6A1	ITGB1	Fibro 5	Fibro 5
COL6A2_ITGB1	COL6A2	ITGB1	Fibro 5	Fibro 5
COL6A3_ITGB1	COL6A3	ITGB1	Fibro 5	Fibro 5
FBLN1_ITGB1	FBLN1	ITGB1	Fibro 5	Fibro 5
FBN1_ITGB1	FBN1	ITGB1	Fibro 5	Fibro 5
FN1_ITGB1	FN1	ITGB1	Fibro 5	Fibro 5
HSPG2_ITGB1	HSPG2	ITGB1	Fibro 5	Fibro 5
HSPG2_LRP1	HSPG2	LRP1	Fibro 5	Fibro 5
LAMA2_ITGB1	LAMA2	ITGB1	Fibro 5	Fibro 5
LAMC1_ITGB1	LAMC1	ITGB1	Fibro 5	Fibro 5
LGALS1_ITGB1	LGALS1	ITGB1	Fibro 5	Fibro 5
LUM_ITGB1	LUM	ITGB1	Fibro 5	Fibro 5
MFGE8_PDGFRB	MFGE8	PDGFRB	Fibro 5	Fibro 5
PSAP_LRP1	PSAP	LRP1	Fibro 5	Fibro 5
SERPINE2_LRP1	SERPINE2	LRP1	Fibro 5	Fibro 5
SERPING1_LRP1	SERPING1	LRP1	Fibro 5	Fibro 5
TFPI_LRP1	TFPI	LRP1	Fibro 5	Fibro 5
TIMP1_CD63	TIMP1	CD63	Fibro 5	Fibro 5
VCAN_ITGB1	VCAN	ITGB1	Fibro 5	Fibro 5
APP_LRP1	APP	LRP1	Fibro 5	Fibro 6
COL1A1_ITGB1	COL1A1	ITGB1	Fibro 5	Fibro 6
COL1A2_ITGB1	COL1A2	ITGB1	Fibro 5	Fibro 6
COL3A1_ITGB1	COL3A1	ITGB1	Fibro 5	Fibro 6
COL6A1_ITGB1	COL6A1	ITGB1	Fibro 5	Fibro 6
COL6A2_ITGB1	COL6A2	ITGB1	Fibro 5	Fibro 6
COL6A3_ITGB1	COL6A3	ITGB1	Fibro 5	Fibro 6
FBLN1_ITGB1	FBLN1	ITGB1	Fibro 5	Fibro 6
FBN1_ITGB1	FBN1	ITGB1	Fibro 5	Fibro 6
FN1_ITGB1	FN1	ITGB1	Fibro 5	Fibro 6
HSPG2_ITGB1	HSPG2	ITGB1	Fibro 5	Fibro 6
HSPG2_LRP1	HSPG2	LRP1	Fibro 5	Fibro 6
LAMA2_ITGB1	LAMA2	ITGB1	Fibro 5	Fibro 6
LAMC1_ITGB1	LAMC1	ITGB1	Fibro 5	Fibro 6
LGALS1_ITGB1	LGALS1	ITGB1	Fibro 5	Fibro 6
LUM_ITGB1	LUM	ITGB1	Fibro 5	Fibro 6
MFGE8_PDGFRB	MFGE8	PDGFRB	Fibro 5	Fibro 6
PSAP_LRP1	PSAP	LRP1	Fibro 5	Fibro 6
SERPINE2_LRP1	SERPINE2	LRP1	Fibro 5	Fibro 6
SERPING1_LRP1	SERPING1	LRP1	Fibro 5	Fibro 6
TFPI_LRP1	TFPI	LRP1	Fibro 5	Fibro 6
TIMP1_CD63	TIMP1	CD63	Fibro 5	Fibro 6
VCAN_ITGB1	VCAN	ITGB1	Fibro 5	Fibro 6

Non-obstructive				
Pair_Name	Ligand	Receptor	L_cell	R_cell
A2M_LRP1	A2M	LRP1	Fibro 2	Fibro 2
CALM1_RYR2	CALM1	RYR2	Fibro 2	Fibro 2
CCN2_LRP1	CCN2	LRP1	Fibro 2	Fibro 2
COL1A1_CD36	COL1A1	CD36	Fibro 2	Fibro 2
COL1A1_ITGB1	COL1A1	ITGB1	Fibro 2	Fibro 2
COL1A2_CD36	COL1A2	CD36	Fibro 2	Fibro 2
COL1A2_ITGB1	COL1A2	ITGB1	Fibro 2	Fibro 2
COL3A1_ITGB1	COL3A1	ITGB1	Fibro 2	Fibro 2
COL4A1_ITGB1	COL4A1	ITGB1	Fibro 2	Fibro 2
COL6A1_ITGB1	COL6A1	ITGB1	Fibro 2	Fibro 2
COL6A2_ITGB1	COL6A2	ITGB1	Fibro 2	Fibro 2
COL6A3_ITGB1	COL6A3	ITGB1	Fibro 2	Fibro 2
FN1_ITGB1	FN1	ITGB1	Fibro 2	Fibro 2
HSP90AA1_LRP1	HSP90AA1	LRP1	Fibro 2	Fibro 2
HSP90B1_LRP1	HSP90B1	LRP1	Fibro 2	Fibro 2
LAMA2_ITGB1	LAMA2	ITGB1	Fibro 2	Fibro 2
LAMB1_ITGB1	LAMB1	ITGB1	Fibro 2	Fibro 2
LGALS1_ITGB1	LGALS1	ITGB1	Fibro 2	Fibro 2
LPL_LRP1	LPL	LRP1	Fibro 2	Fibro 2
LUM_ITGB1	LUM	ITGB1	Fibro 2	Fibro 2
PSAP_LRP1	PSAP	LRP1	Fibro 2	Fibro 2
S100A1_RYR2	S100A1	RYR2	Fibro 2	Fibro 2
TIMP1_CD63	TIMP1	CD63	Fibro 2	Fibro 2
TIMP2_ITGB1	TIMP2	ITGB1	Fibro 2	Fibro 2
VCAN_ITGB1	VCAN	ITGB1	Fibro 2	Fibro 2
CALM1_RYR2	CALM1	RYR2	Fibro 2	Fibro 3
COL1A1_CD36	COL1A1	CD36	Fibro 2	Fibro 3
COL1A1_ITGB1	COL1A1	ITGB1	Fibro 2	Fibro 3
COL1A2_CD36	COL1A2	CD36	Fibro 2	Fibro 3
COL1A2_ITGB1	COL1A2	ITGB1	Fibro 2	Fibro 3
COL3A1_ITGB1	COL3A1	ITGB1	Fibro 2	Fibro 3
COL4A1_ITGB1	COL4A1	ITGB1	Fibro 2	Fibro 3
COL6A1_ITGB1	COL6A1	ITGB1	Fibro 2	Fibro 3
COL6A2_ITGB1	COL6A2	ITGB1	Fibro 2	Fibro 3
COL6A3_ITGB1	COL6A3	ITGB1	Fibro 2	Fibro 3
FN1_ITGB1	FN1	ITGB1	Fibro 2	Fibro 3
LAMA2_ITGB1	LAMA2	ITGB1	Fibro 2	Fibro 3
LAMB1_ITGB1	LAMB1	ITGB1	Fibro 2	Fibro 3
LGALS1_ITGB1	LGALS1	ITGB1	Fibro 2	Fibro 3
LUM_ITGB1	LUM	ITGB1	Fibro 2	Fibro 3
S100A1_RYR2	S100A1	RYR2	Fibro 2	Fibro 3
TIMP1_CD63	TIMP1	CD63	Fibro 2	Fibro 3
TIMP2_ITGB1	TIMP2	ITGB1	Fibro 2	Fibro 3
VCAN_ITGB1	VCAN	ITGB1	Fibro 2	Fibro 3
CALM1_RYR2	CALM1	RYR2	Fibro 2	Fibro 4
COL1A1_CD36	COL1A1	CD36	Fibro 2	Fibro 4
COL1A1_ITGB1	COL1A1	ITGB1	Fibro 2	Fibro 4
COL1A2_CD36	COL1A2	CD36	Fibro 2	Fibro 4
COL1A2_ITGB1	COL1A2	ITGB1	Fibro 2	Fibro 4
COL3A1_ITGB1	COL3A1	ITGB1	Fibro 2	Fibro 4
COL4A1_ITGB1	COL4A1	ITGB1	Fibro 2	Fibro 4
COL6A1_ITGB1	COL6A1	ITGB1	Fibro 2	Fibro 4
COL6A2_ITGB1	COL6A2	ITGB1	Fibro 2	Fibro 4
COL6A3_ITGB1	COL6A3	ITGB1	Fibro 2	Fibro 4
FN1_ITGB1	FN1	ITGB1	Fibro 2	Fibro 4
LAMA2_ITGB1	LAMA2	ITGB1	Fibro 2	Fibro 4
LAMB1_ITGB1	LAMB1	ITGB1	Fibro 2	Fibro 4
LGALS1_ITGB1	LGALS1	ITGB1	Fibro 2	Fibro 4
LUM_ITGB1	LUM	ITGB1	Fibro 2	Fibro 4
S100A1_RYR2	S100A1	RYR2	Fibro 2	Fibro 4
TIMP1_CD63	TIMP1	CD63	Fibro 2	Fibro 4
TIMP2_ITGB1	TIMP2	ITGB1	Fibro 2	Fibro 4
VCAN_ITGB1	VCAN	ITGB1	Fibro 2	Fibro 4
CALM1_RYR2	CALM1	RYR2	Fibro 2	Fibro 5
COL1A1_CD36	COL1A1	CD36	Fibro 2	Fibro 5
COL1A1_ITGB1	COL1A1	ITGB1	Fibro 2	Fibro 5
COL1A2_CD36	COL1A2	CD36	Fibro 2	Fibro 5
COL1A2_ITGB1	COL1A2	ITGB1	Fibro 2	Fibro 5
COL3A1_ITGB1	COL3A1	ITGB1	Fibro 2	Fibro 5
COL4A1_ITGB1	COL4A1	ITGB1	Fibro 2	Fibro 5
COL6A1_ITGB1	COL6A1	ITGB1	Fibro 2	Fibro 5
COL6A2_ITGB1	COL6A2	ITGB1	Fibro 2	Fibro 5
COL6A3_ITGB1	COL6A3	ITGB1	Fibro 2	Fibro 5
FN1_ITGB1	FN1	ITGB1	Fibro 2	Fibro 5
LAMA2_ITGB1	LAMA2	ITGB1	Fibro 2	Fibro 5
LAMB1_ITGB1	LAMB1	ITGB1	Fibro 2	Fibro 5
LGALS1_ITGB1	LGALS1	ITGB1	Fibro 2	Fibro 5
LUM_ITGB1	LUM	ITGB1	Fibro 2	Fibro 5
S100A1_RYR2	S100A1	RYR2	Fibro 2	Fibro 5
TIMP1_CD63	TIMP1	CD63	Fibro 2	Fibro 5
TIMP2_ITGB1	TIMP2	ITGB1	Fibro 2	Fibro 5
VCAN_ITGB1	VCAN	ITGB1	Fibro 2	Fibro 5
CALM1_RYR2	CALM1	RYR2	Fibro 2	Fibro 6
COL1A1_CD36	COL1A1	CD36	Fibro 2	Fibro 6
COL1A1_ITGB1	COL1A1	ITGB1	Fibro 2	Fibro 6
COL1A2_CD36	COL1A2	CD36	Fibro 2	Fibro 6
COL1A2_ITGB1	COL1A2	ITGB1	Fibro 2	Fibro 6
COL3A1_ITGB1	COL3A1	ITGB1	Fibro 2	Fibro 6
COL4A1_ITGB1	COL4A1	ITGB1	Fibro 2	Fibro 6
COL6A1_ITGB1	COL6A1	ITGB1	Fibro 2	Fibro 6
COL6A2_ITGB1	COL6A2	ITGB1	Fibro 2	Fibro 6
COL6A3_ITGB1	COL6A3	ITGB1	Fibro 2	Fibro 6
FN1_ITGB1	FN1	ITGB1	Fibro 2	Fibro 6
LAMA2_ITGB1	LAMA2	ITGB1	Fibro 2	Fibro 6
LAMB1_ITGB1	LAMB1	ITGB1	Fibro 2	Fibro 6
LGALS1_ITGB1	LGALS1	ITGB1	Fibro 2	Fibro 6
LUM_ITGB1	LUM	ITGB1	Fibro 2	Fibro 6
S100A1_RYR2	S100A1	RYR2	Fibro 2	Fibro 6
TIMP1_CD63	TIMP1	CD63	Fibro 2	Fibro 6
TIMP2_ITGB1	TIMP2	ITGB1	Fibro 2	Fibro 6
VCAN_ITGB1	VCAN	ITGB1	Fibro 2	Fibro 6
A2M_LRP1	A2M	LRP1	Fibro 5	Fibro 2
CALM1_RYR2	CALM1	RYR2	Fibro 5	Fibro 2
FN1_ITGB1	FN1	ITGB1	Fibro 5	Fibro 2
HSP90AA1_LRP1	HSP90AA1	LRP1	Fibro 5	Fibro 2
LPL_LRP1	LPL	LRP1	Fibro 5	Fibro 2
LUM_ITGB1	LUM	ITGB1	Fibro 5	Fibro 2
PSAP_LRP1	PSAP	LRP1	Fibro 5	Fibro 2
S100A1_RYR2	S100A1	RYR2	Fibro 5	Fibro 2
CALM1_RYR2	CALM1	RYR2	Fibro 5	Fibro 3
FN1_ITGB1	FN1	ITGB1	Fibro 5	Fibro 3
LUM_ITGB1	LUM	ITGB1	Fibro 5	Fibro 3
S100A1_RYR2	S100A1	RYR2	Fibro 5	Fibro 3
CALM1_RYR2	CALM1	RYR2	Fibro 5	Fibro 4
FN1_ITGB1	FN1	ITGB1	Fibro 5	Fibro 4
LUM_ITGB1	LUM	ITGB1	Fibro 5	Fibro 4
S100A1_RYR2	S100A1	RYR2	Fibro 5	Fibro 4
CALM1_RYR2	CALM1	RYR2	Fibro 5	Fibro 5
FN1_ITGB1	FN1	ITGB1	Fibro 5	Fibro 5
LUM_ITGB1	LUM	ITGB1	Fibro 5	Fibro 5
S100A1_RYR2	S100A1	RYR2	Fibro 5	Fibro 5
CALM1_RYR2	CALM1	RYR2	Fibro 5	Fibro 6
FN1_ITGB1	FN1	ITGB1	Fibro 5	Fibro 6
LUM_ITGB1	LUM	ITGB1	Fibro 5	Fibro 6
S100A1_RYR2	S100A1	RYR2	Fibro 5	Fibro 6

Supplemental Table 14. Increased Communication From Fibroblast and Cardiomyocyte Subtypes to Cardiomyocyte Cluster 9

Normal				
**Pair_Name**	**Ligand**	**Receptor**	**L_cell**	**R_cell**
COL1A1_CD36	COL1A1	CD36	Fibro 1	CM 9
COL1A2_CD36	COL1A2	CD36	Fibro 1	CM 9
TIMP1_CD63	TIMP1	CD63	Fibro 1	CM 9
COL1A1_CD36	COL1A1	CD36	Fibro 2	CM 9
COL1A2_CD36	COL1A2	CD36	Fibro 2	CM 9
THBS2_CD36	THBS2	CD36	Fibro 2	CM 9
TIMP1_CD63	TIMP1	CD63	Fibro 2	CM 9
COL1A2_CD36	COL1A2	CD36	Fibro 3	CM 9
TIMP1_CD63	TIMP1	CD63	Fibro 3	CM 9
COL1A1_CD36	COL1A1	CD36	Fibro 4	CM 9
COL1A2_CD36	COL1A2	CD36	Fibro 4	CM 9
TIMP1_CD63	TIMP1	CD63	Fibro 4	CM 9
COL1A1_CD36	COL1A1	CD36	Fibro 5	CM 9
COL1A2_CD36	COL1A2	CD36	Fibro 5	CM 9
TIMP1_CD63	TIMP1	CD63	Fibro 5	CM 9
COL1A2_CD36	COL1A2	CD36	CM 2	CM 9
TIMP1_CD63	TIMP1	CD63	CM 2	CM 9
COL1A2_CD36	COL1A2	CD36	CM 3	CM 9
TIMP1_CD63	TIMP1	CD63	CM 3	CM 9
CALM1_RYR2	CALM1	RYR2	CM 5	CM 9
COL1A2_CD36	COL1A2	CD36	CM 5	CM 9
S100A1_RYR2	S100A1	RYR2	CM 5	CM 9
TIMP1_CD63	TIMP1	CD63	CM 5	CM 9

Non-obstructive				
**Pair_Name**	**Ligand**	**Receptor**	**L_cell**	**R_cell**
CALM1_CACNA1C	CALM1	CACNA1C	Fibro 1	CM 9
CALM1_PDE1C	CALM1	PDE1C	Fibro 1	CM 9
CALM1_RYR2	CALM1	RYR2	Fibro 1	CM 9
COL1A2_CD36	COL1A2	CD36	Fibro 1	CM 9
COL1A2_ITGB1	COL1A2	ITGB1	Fibro 1	CM 9
COL4A1_ITGB1	COL4A1	ITGB1	Fibro 1	CM 9
COL6A1_ITGB1	COL6A1	ITGB1	Fibro 1	CM 9
COL6A2_ITGB1	COL6A2	ITGB1	Fibro 1	CM 9
COL6A3_ITGB1	COL6A3	ITGB1	Fibro 1	CM 9
FN1_ITGB1	FN1	ITGB1	Fibro 1	CM 9
LAMA2_ITGB1	LAMA2	ITGB1	Fibro 1	CM 9
LGALS1_ITGB1	LGALS1	ITGB1	Fibro 1	CM 9
LUM_ITGB1	LUM	ITGB1	Fibro 1	CM 9
S100A1_RYR2	S100A1	RYR2	Fibro 1	CM 9
CALM1_CACNA1C	CALM1	CACNA1C	Fibro 2	CM 9
CALM1_PDE1C	CALM1	PDE1C	Fibro 2	CM 9
CALM1_RYR2	CALM1	RYR2	Fibro 2	CM 9
CALM2_CACNA1C	CALM2	CACNA1C	Fibro 2	CM 9
CALM2_PDE1C	CALM2	PDE1C	Fibro 2	CM 9
COL1A1_CD36	COL1A1	CD36	Fibro 2	CM 9
COL1A1_ITGB1	COL1A1	ITGB1	Fibro 2	CM 9
COL1A2_CD36	COL1A2	CD36	Fibro 2	CM 9
COL1A2_ITGB1	COL1A2	ITGB1	Fibro 2	CM 9
COL3A1_ITGB1	COL3A1	ITGB1	Fibro 2	CM 9
COL4A1_ITGB1	COL4A1	ITGB1	Fibro 2	CM 9
COL6A1_ITGB1	COL6A1	ITGB1	Fibro 2	CM 9
COL6A2_ITGB1	COL6A2	ITGB1	Fibro 2	CM 9
COL6A3_ITGB1	COL6A3	ITGB1	Fibro 2	CM 9
FN1_ITGB1	FN1	ITGB1	Fibro 2	CM 9
LAMA2_ITGB1	LAMA2	ITGB1	Fibro 2	CM 9
LAMB1_ITGB1	LAMB1	ITGB1	Fibro 2	CM 9
LGALS1_ITGB1	LGALS1	ITGB1	Fibro 2	CM 9
LUM_ITGB1	LUM	ITGB1	Fibro 2	CM 9
S100A1_RYR2	S100A1	RYR2	Fibro 2	CM 9
TIMP1_CD63	TIMP1	CD63	Fibro 2	CM 9
TIMP2_ITGB1	TIMP2	ITGB1	Fibro 2	CM 9
VCAN_ITGB1	VCAN	ITGB1	Fibro 2	CM 9
CALM1_CACNA1C	CALM1	CACNA1C	Fibro 3	CM 9
CALM1_PDE1C	CALM1	PDE1C	Fibro 3	CM 9
CALM1_RYR2	CALM1	RYR2	Fibro 3	CM 9
COL1A2_CD36	COL1A2	CD36	Fibro 3	CM 9
COL1A2_ITGB1	COL1A2	ITGB1	Fibro 3	CM 9
COL3A1_ITGB1	COL3A1	ITGB1	Fibro 3	CM 9
COL4A1_ITGB1	COL4A1	ITGB1	Fibro 3	CM 9
COL6A1_ITGB1	COL6A1	ITGB1	Fibro 3	CM 9
COL6A2_ITGB1	COL6A2	ITGB1	Fibro 3	CM 9
COL6A3_ITGB1	COL6A3	ITGB1	Fibro 3	CM 9
FN1_ITGB1	FN1	ITGB1	Fibro 3	CM 9
LAMA2_ITGB1	LAMA2	ITGB1	Fibro 3	CM 9
LAMB1_ITGB1	LAMB1	ITGB1	Fibro 3	CM 9
LGALS1_ITGB1	LGALS1	ITGB1	Fibro 3	CM 9
LUM_ITGB1	LUM	ITGB1	Fibro 3	CM 9
S100A1_RYR2	S100A1	RYR2	Fibro 3	CM 9
CALM1_CACNA1C	CALM1	CACNA1C	Fibro 4	CM 9
CALM1_PDE1C	CALM1	PDE1C	Fibro 4	CM 9
CALM1_RYR2	CALM1	RYR2	Fibro 4	CM 9
COL1A2_CD36	COL1A2	CD36	Fibro 4	CM 9
COL1A2_ITGB1	COL1A2	ITGB1	Fibro 4	CM 9
COL4A1_ITGB1	COL4A1	ITGB1	Fibro 4	CM 9
COL6A1_ITGB1	COL6A1	ITGB1	Fibro 4	CM 9
COL6A3_ITGB1	COL6A3	ITGB1	Fibro 4	CM 9
FN1_ITGB1	FN1	ITGB1	Fibro 4	CM 9
LGALS1_ITGB1	LGALS1	ITGB1	Fibro 4	CM 9
LUM_ITGB1	LUM	ITGB1	Fibro 4	CM 9
S100A1_RYR2	S100A1	RYR2	Fibro 4	CM 9
CALM1_CACNA1C	CALM1	CACNA1C	Fibro 5	CM 9
CALM1_PDE1C	CALM1	PDE1C	Fibro 5	CM 9
CALM1_RYR2	CALM1	RYR2	Fibro 5	CM 9
FN1_ITGB1	FN1	ITGB1	Fibro 5	CM 9
LUM_ITGB1	LUM	ITGB1	Fibro 5	CM 9
S100A1_RYR2	S100A1	RYR2	Fibro 5	CM 9
CALM1_CACNA1C	CALM1	CACNA1C	CM 2	CM 9
CALM1_PDE1C	CALM1	PDE1C	CM 2	CM 9
CALM1_RYR2	CALM1	RYR2	CM 2	CM 9
CALM2_CACNA1C	CALM2	CACNA1C	CM 2	CM 9
CALM2_PDE1C	CALM2	PDE1C	CM 2	CM 9
COL1A2_CD36	COL1A2	CD36	CM 2	CM 9
COL1A2_ITGB1	COL1A2	ITGB1	CM 2	CM 9
COL6A2_ITGB1	COL6A2	ITGB1	CM 2	CM 9
FN1_ITGB1	FN1	ITGB1	CM 2	CM 9
LGALS1_ITGB1	LGALS1	ITGB1	CM 2	CM 9
LUM_ITGB1	LUM	ITGB1	CM 2	CM 9
S100A1_RYR2	S100A1	RYR2	CM 2	CM 9
TIMP1_CD63	TIMP1	CD63	CM 2	CM 9
CALM1_CACNA1C	CALM1	CACNA1C	CM 3	CM 9
CALM1_PDE1C	CALM1	PDE1C	CM 3	CM 9
CALM1_RYR2	CALM1	RYR2	CM 3	CM 9
CALM2_CACNA1C	CALM2	CACNA1C	CM 3	CM 9
CALM2_PDE1C	CALM2	PDE1C	CM 3	CM 9
LAMA2_ITGB1	LAMA2	ITGB1	CM 3	CM 9
LGALS1_ITGB1	LGALS1	ITGB1	CM 3	CM 9
NCAM1_CACNA1C	NCAM1	CACNA1C	CM 3	CM 9
S100A1_RYR2	S100A1	RYR2	CM 3	CM 9
TGM2_ITGB1	TGM2	ITGB1	CM 3	CM 9
VEGFA_ITGB1	VEGFA	ITGB1	CM 3	CM 9
CALM1_CACNA1C	CALM1	CACNA1C	CM 5	CM 9
CALM1_PDE1C	CALM1	PDE1C	CM 5	CM 9
CALM1_RYR2	CALM1	RYR2	CM 5	CM 9
CALM2_CACNA1C	CALM2	CACNA1C	CM 5	CM 9
CALM2_PDE1C	CALM2	PDE1C	CM 5	CM 9
CALM3_CACNA1C	CALM3	CACNA1C	CM 5	CM 9
CALM3_PDE1C	CALM3	PDE1C	CM 5	CM 9
CALM3_RYR2	CALM3	RYR2	CM 5	CM 9
COL1A2_CD36	COL1A2	CD36	CM 5	CM 9
COL1A2_ITGB1	COL1A2	ITGB1	CM 5	CM 9
COL6A1_ITGB1	COL6A1	ITGB1	CM 5	CM 9
COL6A2_ITGB1	COL6A2	ITGB1	CM 5	CM 9
FN1_ITGB1	FN1	ITGB1	CM 5	CM 9
LAMB2_ITGB1	LAMB2	ITGB1	CM 5	CM 9
LGALS1_ITGB1	LGALS1	ITGB1	CM 5	CM 9
LGALS3BP_ITGB1	LGALS3BP	ITGB1	CM 5	CM 9
LUM_ITGB1	LUM	ITGB1	CM 5	CM 9
S100A1_RYR2	S100A1	RYR2	CM 5	CM 9
TGM2_ITGB1	TGM2	ITGB1	CM 5	CM 9
TIMP1_CD63	TIMP1	CD63	CM 5	CM 9
